# Endophytic Fungal Diversity in *Cirsium kawakamii* from Taiwan

**DOI:** 10.3390/jof9111076

**Published:** 2023-11-03

**Authors:** Yi-Jeng Chen, Hui-Juan Chen, Wen-Hsin Chung

**Affiliations:** 1 Department of Plant Medicine, National Chiayi University, Chiayi 600, Taiwan; ejchen04@mail.ncyu.edu.tw; 2 Department of Plant Pathology, National Chung Hsing University, Taichung 402, Taiwan; 3 Master Program in Plant Medicine and Good Agricultural Practice, National Chung Hsing University, Taichung 402, Taiwan

**Keywords:** *Cirsium kawakamii*, endophytic fungal diversity, *Fusarium tricinctum* species complex (FTSC), pathogen

## Abstract

The endophytic fungal diversity of *Cirsium kawakamii*, a herb indigenous to Taiwan, was analyzed in this study. In addition, some fungal isolates were evaluated for the risk they pose as plant pathogens. In total, 1836 endophytic fungi were isolated from *C. kawakamii* from Hehuanjian, Puli Township, and Tatachia. They were classified into 2 phyla, 8 classes, 40 families, and 68 genera. *Colletotrichum*, *Fusarium*, *Phomopsis*, and *Xylaria*, (Ascomycota, Sordariomycetes) were the dominant genera. The genus accumulation curve (based on the bootstrap estimator) was non-asymptotic, with estimated richness significantly exceeding the richness captured by our sampling to date. Considering the collection time, the data indicated significant differences in the proportions of the *C. kawakamii* endophyte genus from Hehuanjan, Puli Township (across two seasons), and Tatachia. The Shannon and Gini–Simpson indices revealed variations in diversity, with *C. kawakamii* endophytes (Puli Township in winter) significantly reducing alpha diversity compared with other seasons and locations. Meanwhile, the Gini–Simpson index suggested that there were no significant differences in richness among the four sampling sites. The PCA results unveiled distinct community structures across different locations and seasons, explaining 46.73% of the total variation in fungal community composition significantly affected diversity and richness. In addition, a considerable number of *Fusarium* isolates exhibited harmful properties towards wheat, potatoes, and apples. It is postulated that these fungi belong to the *Fusarium tricinctum* species complex (FTSC).

## 1. Introduction

Endophytes are microbes that colonize or infect the living internal tissues of a host plant without causing symptoms [1,2]. Endophytes have been found in almost all tested plants, and symbiosis between plants and endophytes is widespread in natural ecosystems [3,4]. Several fossil records reveal that symbiosis may have evolved over the last 400 million years [5]. Symbionts play essential roles in plant metabolism and development on a microscopic scale, and endophytes profoundly affect plant fitness, ecology, evolution, and communities on a macroscopic scale [6,7]. As such, endophytes are often regarded as mutualistic with their host plants, and their beneficial effects depend on the species, behavior, life strategy, and physiological status of both the hosts and endophytes [8].

Endophytes are considered rich biological resources in agriculture, medicine, and the food industry [9,10]. They can produce bioactive compounds similar or identical to those originating from their host plants [11]. For example, *Taxomyces andreanae*, an endophyte isolated from *Taxus brevifolia*, can produce taxol, which has anticancer activity [12]. Several endophytes, such as *Fusarium solani*, *F. lateritium*, *F. mairie*, *Pestalotiopsis microspora*, and *Perconia* sp., can synthesize taxol and its derivatives [13]. The secondary metabolite sordaricin, with antifungal activity, is produced by the endophytic *Xylaria* [14]. Nonetheless, some endophytes are aggressive saprophytes or pose a risk as opportunistic pathogens [15]. Exploring their roles in natural environments or host plants by examining their diversity will improve our understanding of endophytes as beneficial resources.

Endophytes are believed to have coevolved with their host plants, and certain microorganisms are frequently found in specific plant populations [16]. Endophytic organisms can be transmitted horizontally and can establish symbiotic relationships with their host plants after contact with vectors such as water and air. Endophytic diversity and abundance are influenced by the host plant’s geographic location [17]. The composition, diversity, and abundance of endophytic fungi in different locations, environments, and crop species, as well as the influence of various factors, have been widely analyzed [18,19,20,21,22,23]. However, owing to factors such as plant diversity, climate variability, the complex interconnectedness between plant distribution and ecosystems, and the cryptic nature of endophytic fungi, more research is needed to understand the relationships between endophytic fungi and plants [17].

*Cirsium kawakamii* Hayata (Asteraceae) is a plant endemic to Taiwan [24]. *Cirsium* aids in liver protection and may become an important biological resource. *Cirsium kawakamii* contains several hepatoprotective compounds, including silibinin α, silibinin β, and caffeoylquinic acid [25,26]. Although certain *Cirsium* species are believed to have a rich endophytic bacterial community [27], the diversity of endophytic fungi in *C. kawakamii* remains unclear. During our preliminary research, we identified various endophytic fungi on *C. kawakamii*, some potential plant pathogens. Thus, the aims of this study are: (1) to investigate the endophytic fungal diversity of *C. kawakamii* and (2) to evaluate the risk of some endophytic fungal isolates as plant pathogens.

## 2. Materials and Methods

### 2.1. Plant Material and Endophytic Fungi 

**Figure 1:**
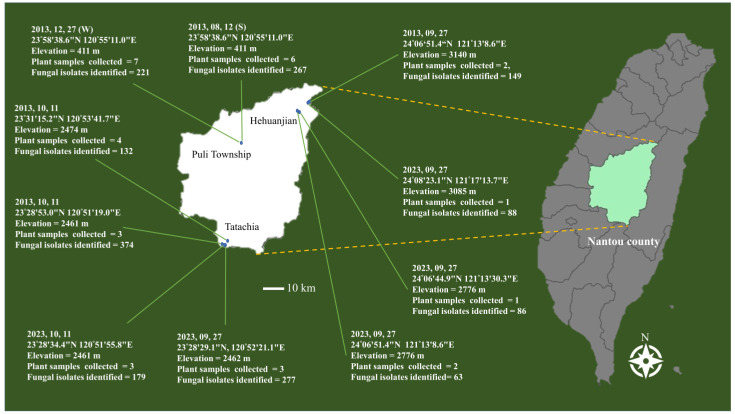
*Cirsium kawakamii* collection sites and number of endophytic fungal isolates identified.

*Cirsium kawakamii* is a high-altitude plant. Thirty-two healthy entire plants of *C. kawakamii* were collected from one site in Puli Township (during two seasons), four sites in Hehuanjian, and four sites in Tatachia between August and December 2013 (Figure 1). The method for isolating endophytic fungi was a modified version of that described by Bill et al. [28]. Leaf veins and stem tissue of the plant samples were washed with reverse osmosis water, cut into segments (3–5 cm), and surface-sterilized with 75% ethanol for 1 min. Next, they were soaked in a 1% sodium hypochlorite solution (NaOCl) for 7 min and washed three times with sterile water. Sterilized tissue segments were further cut into fragments (1–3 cm), which were then placed on potato sucrose agar (PSA) medium in Petri dishes and incubated at 25 °C in the dark. After 3–5 d of incubation, the fungi that grew from the segments were transferred onto PSA, and each fungus was purified via single hyphal tip isolation. Pure fungal cultures were stored at −80 °C in 10% glycerol diluted in sterile water.

### 2.2. Morphological and Molecular Identification of Endophytic Fungi

The purified endophytic fungi were cultured in PSA medium at 25 °C with 12 h light exposure. After 14 d of cultivation, the colonies were preliminarily classified based on colony pattern morphology, and 10% of the strains were selected from each group for molecular identification. Total DNA was extracted using a modified method published by Goodwin and Lee [29]. This involved adding an appropriate amount of hyphae to a lysis solution and freezing the hyphae at −20 °C. The hyphae were then crushed and kept at 65 °C for 30 min. DNA was then extracted using phenol/chloroform, precipitated with isopropanol, washed with ethanol, and dried with alcohol. The DNA was dissolved in deionized water (Milli-Q water) at 65 °C for later use. The rDNA internal transcribed spacer (ITS) region was amplified via PCR using the primer pair ITS1 (5′-CTTGGTCATTTAGAGGAAGTAA-3′)/ITS4 (5′-TCCTCCGCTTATTGATATGC-3′) [30]. The PCR reaction conditions for ITS rDNA were 95 °C for 2 min, followed by 35 cycles of 95 °C for 30 s, 55 °C for 30 s, and 72 °C for 120 s, with a final extension at 72 °C for 5 min. The PCR products were sent to Tri-I Biotech, Inc. (Taichung, Taiwan) for sequencing. The sequences were aligned using the online tools at the National Center for Biotechnology Information (NCBI) website (http://www.ncbi.nlm.nih.gov/ (accessed on 15 March 2015)).

### 2.3. Alpha and Beta Diversity Analysis of Endophytic Fungi

Estimate S 9.1 (http://viceroy.eeb.uconn.edu/estimates/index.html (accessed on 13 April 2015)) was used to analyze endophytic fungal diversity, species richness, and similarity. Shannon diversity, Chao1 richness, and Gini–Simpson indices were selected based on genus level to assess the alpha diversity of endophytic fungi to *C. kawakamii* in different sampling areas in Nantou County, Taiwan. In addition, bootstrap richness estimators predicted the number of genera present in the endophytic fungal community of *C. kawakamii* and the genus accumulation curve drawn based on the bootstrap estimator. Beta diversity was estimated using PAST 4.03 software according to Bray–Curtis distances across endophytic fungal flora in plant samples in habitats. Principal component analysis (PCA) was performed in the IBM SPSS 20.0 software.

The differences in the alpha diversity index of endophytic fungal flora among the sampling areas were tested by a one-way analysis of variance (ANOVA). A Tukey test was used to determine whether differences between means were statistically significant. In all tests, *p*-value < 0.05 was considered statistically significant.

### 2.4. Identification of Phytopathogen-like Endophytic Fusarium sp. Isolates

Preliminary analysis indicated that the several endophytic *Fusarium* sp. isolates, isolated here, were like the *Fusarium tricinctum* species complex (FTSC) and might be plant pathogens (Table S1). These isolates required further identification and evaluation of their pathogenicity in possible hosts. Identification involved morphological and molecular methods. For morphological observations, these fungal isolates were cultured on synthetic nutrient agar (SNA) medium (1 L sterilized water containing 0.2 g sucrose, 0.2 g glucose, 1.0 g KNO_3_, 1.0 g KH_2_PO_4_, 0.5 g MgSO_4_·7H_2_O, 0.5 g KCl, and 15 g agar) for 10–14 d. The sporulation structures, macroconidia, and microconidia were selected and examined under an optical microscope. Additionally, these fungal isolates were cultured on potato dextrose agar (PDA) for 14–21 d. The mycelium was washed with 5 mL of sterile water. Soil extract broth (SB) was prepared by adding 125 g soil to 500 mL of reverse osmosis (RO) water, followed by shaking at 50 rpm for 2 h, and filtering through a sieve (pore size 0.045 mm); 50 mL of the supernatant was transferred to a 125 mL Erlenmeyer flask, supplemented with 0.025 g dextrose, and autoclaved. The washed mycelium was then added to the flask containing 50 mL soil extract broth and incubated at room temperature with shaking at 50–60 rpm for at least 7 d. The chlamydospores were observed under an optical microscope. Microscope image measurement software (AxioVision Rel. 4.8, Carl Zeiss, Singapore) was used to measure the length, width, and septation of macroconidia, microconidia, and chlamydospores; 120 chlamydospores, 120 microconidia, and 160 macroconidia of each isolate were measured.

Molecular biological methods were used to amplify the total DNA of these Fusarium isolates for nucleotide amplification of ITS rDNA and translation elongation factor subunit alpha (EF1-α), followed by sequence analysis via sequence alignment. ITS rDNA amplification was performed using primers and conditions described in Section 2.2. The EF1-α region was amplified via PCR using the primer pair EF700f (5′-TCTACCAGTGCGGTGGTA-3′)/EF2 (5′-GGA(G/A)GTACCAGT(G/C)ATCATGTT-3′) [31,32]. The PCR reaction conditions were 95 °C for 5 min, followed by 35 cycles of 95 °C for 30 s, 59 °C for 60 s, and 72 °C for 90 s, with a final extension at 72 °C for 10 min. The PCR products were sent to Genomic Information Technology Co., Ltd. (New Taipei, Taiwan) for sequencing, and resulting sequences were aligned using the online NCBI tools. Multiple sequence alignments for the two molecular targets were conducted using the Clustal W algorithm [33] with the MEGA X software (64-bit for Windows), and then the phylogenetic relationships were reconstructed with the same software [34], using the maximum likelihood (ML) method with the combined sequences ITS+EF-1α. The bootstrap method used a heuristic search with 1000 replicates, with random addition of taxa and tree bisection reconnection method. The ITS and EF-1α sequences of *Fusarium tricinctum* species complex (FTSC) reference strains were downloaded from the NCBI database [35]. *Fusarium solani* (strain NRRL 46643) was used as the outgroup. Representative sequences of the ITS and EF-1α of our *Fusarium* isolates were submitted to GenBank; accession numbers are noted in Table S2.

### 2.5. Pathogenicity Test of Endophytic Fusarium Tricinctum Species Complex (FTSC)-like Isolates toward Apple, Potato, and Wheat

Based on a BLAST search with ITS rDNA sequences, 13 endophytic isolates resembling the FTSC-like isolates could potentially be identified as *F. torulosum*, *F. tricinctum*, or *F. flocciferum.* These species are known as the causative agents of potato dry rot, apple fruit rot, and wheat head blight, respectively [36,37,38]. We therefore examined these isolates’ pathogenicity toward these agricultural products.

For apples, the inoculation method was modified according to Sever et al. [39]. Fuji apples (*Malus domestica* Borkh.) were surface-disinfected with 70% ethanol for 30 s, rinsed three times with sterile water, and three wounds 3 mm deep were made with a sterile needle. Endophytic FTSC-like isolate conidial suspensions (2 μL) containing 10^6^ conidia/mL were then inoculated into the wounds. The inoculated apples were placed in a growth chamber at 20 °C with 12 h of light and dark for 28 d. The control was inoculated with SB. Four apples were inoculated with each isolate. The pathogens were re-isolated from the diseased plants and found to be identical to the original isolates. The experiment was performed twice [39].

For potatoes (*Solanum tuberosum* L.), the inoculation method was adapted from Gachango et al. [40]. Conidial suspensions of endophytic FTSC-like isolates (20 μL) containing 105 conidia/mL were inoculated into a 1.5 cm deep wound made with a micropipette tip (2–200 μL pipette tip, DELTALAB^®^, Barcelona, Spain) on a potato tuber. The control group was inoculated with SB. Eight tubers were inoculated with each isolate. After inoculation, four tubers were incubated at 4 °C in the dark for 28 days, while the other four tubers were incubated at 20 °C in the dark for 28 days. Subsequently, disease development was monitored. Pathogens were re-isolated from the diseased plants and found to be identical to the original isolates through morphological and molecular identification methods, confirming the pathogenicity of these endophytic FTSC-like isolates on potatoes. The experiment was conducted twice.

For wheat (*Triticum aestivum* L.), the inoculation method was modified according to Wu et al. [41]. Wheat ‘Tai-Chung 2’ seeds were surface-disinfected with 1% sodium hypochlorite for 1 min, rinsed three times with sterile water, then soaked overnight in water. The seeds were then transferred to a moist filter paper and allowed to germinate at 24 °C for 36 h. The seedlings were then inoculated with endophytic FTSC-like isolates. Wound inoculation was performed by pricking the stem using a sterile needle. Non-wound inoculation was performed by soaking seedlings in endophytic FTSC-like isolate spore suspensions containing 10^5^ conidia/mL for 30 min. Both controls were inoculated with SB. The inoculated seedlings were then placed on a moist filter paper in a growth chamber at 24 °C with 12 h of light for 10 d. Five seeds were inoculated with each isolate, and the pathogens were re-isolated from the diseased plants and found to be identical to the original isolates. The experiment was performed twice.

## 3. Results

### 3.1. Isolation of Endophytic Fungi from Cirsium kawakamii

Between August and December 2013, 32 *Cirsium kawakamii* plant samples were collected from Puli Township, Hehuanjian, and Tatachia. A total of 1836 endophytic fungal isolates were obtained (Table 1). Based on morphological and molecular biology identification, the isolates were classified into 2 phyla, 8 classes (1 uncertain class classification), 40 families (6 uncertain family classifications), and 68 genera. The phyla Ascomycota and Basidiomycota accounted for 99.4% and 0.6% of the isolates, respectively. The classes Sordariomycetes and Dothideomycetes were dominant in Ascomycota, accounting for 81.6% and 14.6% of the isolates, respectively. In Sordariomycetes, the dominant families were Glomerellaceae (monotypic, with only *Colletotrichum*), Xylariaceae, and Nectriaceae, at 30.0%, 23.9%, and 11.2%, respectively. Within the Xylariaceae family, a total of eight genera were identified, which included *Annulohypoxylon*, *Biscogniauxia*, *Daldinia*, *Hypoxylon*, incertae sedis, *Nemania*, *Nodulisporium*, and *Xylaria*. Notably, *Xylaria* stood out as the predominant genus, constituting 17.7%. The family Nectriaceae harbored fungi belonging to two genera: *Fusarium* and *Ilyonectria*. *Fusarium*, in particular, exhibited greater frequency, constituting 11.2% of the total (Table 1). Two Basidiomycota classes were identified: Agaricomycetes and Ustilaginomycetes. In Agaricomycetes, seven isolates (0.3%) and two isolates (0.1%) were identified as Ceratobasidiaceae and Psathyrellaceae, respectively. Additionally, one isolate was consisted as incertae sedis. In Ustilaginomycetes, one family was identified (Ustilaginaceae), and *Pseudozyma* being an isolated genus. In total, 22 fungal isolates could not be conclusively identified. 

### 3.2. Endophytic Fungal Community Composition in the Three Regions

**Figure 2:**
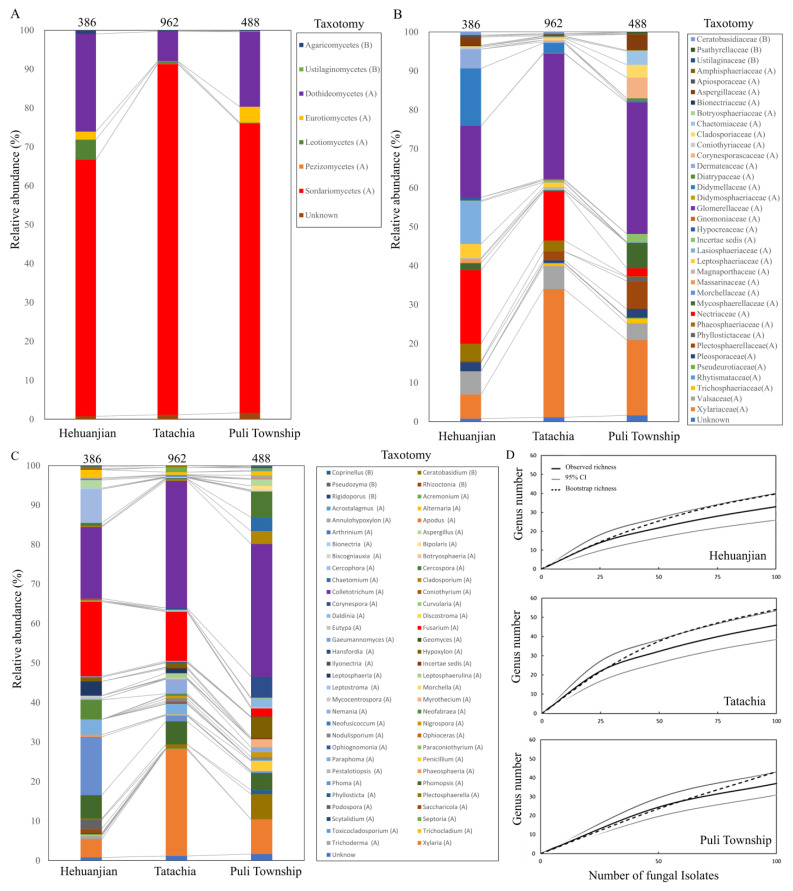
(**A**–**C**) Relative abundance of endophytic fungi in *Cirsium kawakamii* at class, family, and genus levels in different sampling areas. The number at the top of the histogram indicates the number of identified fungi isolates in this analysis. In the taxonomy boxes, (A) = Ascomycota and (B) = Basidiomycota. (**D**) Genus accumulation curve and endophytic fungal composition in *Cirsium kawakamii* from three regions in Taiwan.

The endophytic fungal community composition varied significantly between sampling areas, with significant differences observed in relative abundance at the class, family, and genus levels (*p* < 0.05) (Figure 2A–C). For the samples from Hehuanjian, the endophytic fungal community of *C. kawakamii* comprised 99.0% Ascomycota and 1.0% Basidiomycota. Five classes in Ascomycota were identified: Sordariomycetes, Dothideomycetes, Leotiomycetes, Eurotiomycetes, and incertae sedis, with frequencies of 66.1%, 25.3%, 5.2%, 2.1%, and 0.3%, respectively. Sordariomycetes was dominated by Nectriaceae (with *Fusarium* at 19.1%) and Glomerellaceae (with *Colletotrichum* at 18.3%) (Figure 2B,C).

For the samples from Tatachia, the endophytic fungal community of *C. kawakamii* comprised 99.6% Ascomycota and 0.4% Basidiomycota. Five classes of Ascomycota were identified: Sordariomycetes (91.1%), Dothideomycetes (7.8%), Leotiomycetes (0.5%), Eurotiomycetes (0.1%), and Pezizomycetes (0.1%). In Sordariomycetes, the dominant families were Glomerellaceae (*Colletotrichum*), Xylariaceae, and Nectriaceae, at 32.6%, 33.2%, and 12.7%, respectively. In Xylariaceae, seven genera were identified: *Annulohypoxylon* (0.1%), *Biscogniauxia* (0.1%), *Daldinia* (0.3%), *Hypoxylon* (1.1%), *Nemania* (3.7%), *Nodulisporium* (0.6%), and *Xylaria* (27.3%). In Nectriaceae, *Fusarium* and *Ilyonectria* were identified at 12.6% and 0.1%, respectively (Figure 2B,C).

For the samples from Puli Township, the endophytic fungal community of *C. kawakamii* comprised Ascomycota (99.4%) and Basidiomycota (0.6%). In Ascomycota, four classes were identified: Sordariomycetes (75.4%), Dothideomycetes (19.6%), Leotiomycetes (4.2%), and Eurotiomycetes (0.2%). In Sordariomycetes, Glomerellaceae (*Colletotrichum*) and Xylariaceae were identified, at 34.2% and 19.6%, respectively. Eight genera were identified in Xylariaceae: *Annulohypoxylon* (0.8%), *Biscogniauxia* (0.2%), *Daldinia* (2.1%), *Hypoxylon* (5.2%), incertae sedis (0.2%), *Nemania* (1.3%), *Nodulisporium* (0.8%), and *Xylaria* (9.0%) (Figure 2B,C). The genus accumulation curve drawn based on the bootstrap estimator was not asymptotic (Figure 2D), indicating that we did not isolate all of the endophytic fungal genera present.

### 3.3. Endophytic Fungal Alpha and Beta Diversity in Cirsium kawakamii

**Figure 3:**
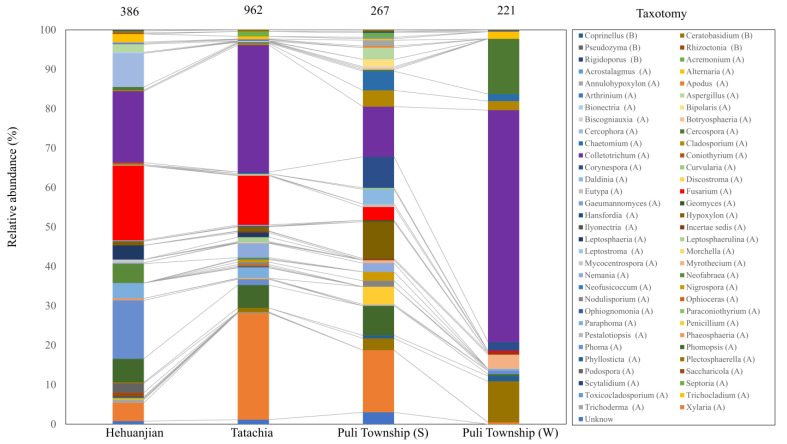
Relative abundance of endophytic fungi of *Cirsium kawakamii* at genus level in different sampling areas (including two seasons in Puli Township; (S) = summer, and (W) = winter). The number at the top of the histogram indicates the number of identified fungi isolates in this analysis. In the taxonomy boxes, (A) = Ascomycota and (B) = Basidiomycota.

**Figure 4:**
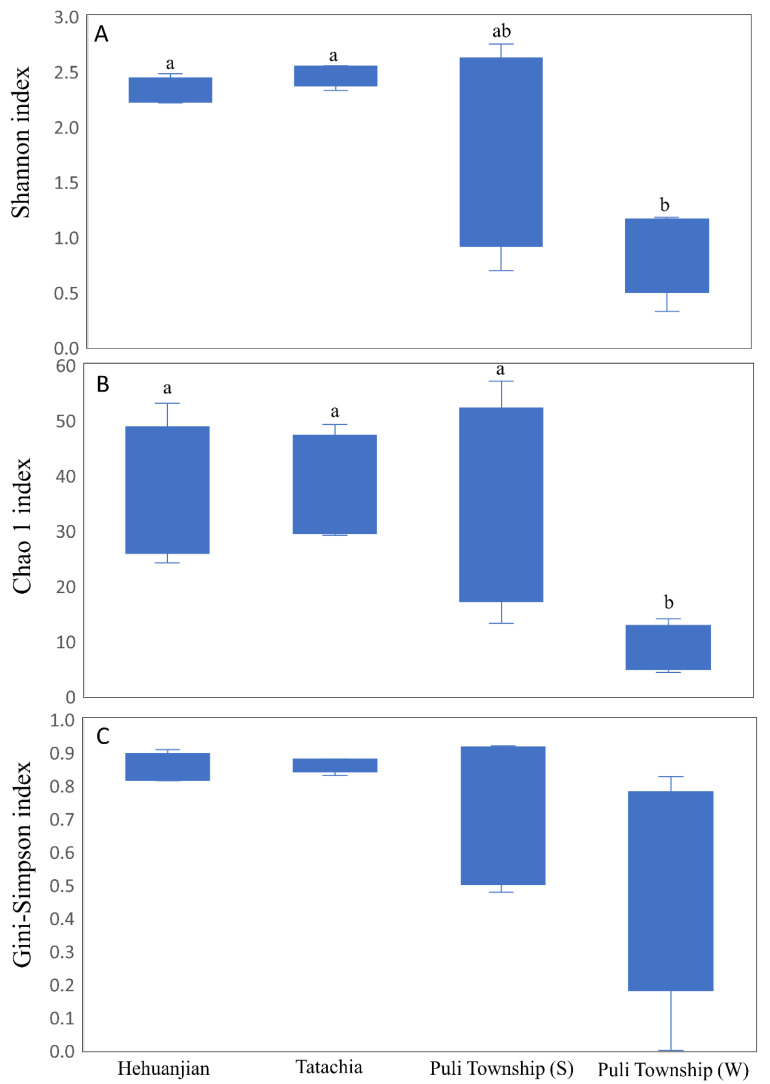
Alpha diversity of *Cirsium kawakamii* endophytic fungal communities: Shannon diversity index (**A**), Chao1 richness (**B**), and Gini–Simpson diversity index (**C**). Letters indicate significant differences in different sampling areas (including two seasons in Puli Township; (S) = summer and (W) = winter) at *p* < 0.05 according to Tukey’s HSD.

When the collection time was considered, the relative abundance map could still observe that endophytes were collected between Hehuanjan, Tatachia, and Puli Township (including samples in two seasons), showing significant differences (*p* < 0.05) in genus proportions (Figure 3). The Shannon and Gini–Simpson indices varied between the study sites. For Hehuanjian, Tatachia, and Puli Townships (two seasons), the Shannon index was 2.31, 2.47, 1.82, and 0.92, respectively, while the Chao 1 index was 36.0, 37.5, 34.1, and 8.5, respectively. (Figure 4A,B). The Shannon and Gini–Simpson indexes indicated *C. kawakamii* endophyte (Puli Township in winter) significantly (*p* < 0.05) decreased the alpha diversity compared with Hehuanjian, Tatachia, and Puli Township (summer). The Gini–Simpson index indicated that there were no significant (*p* < 0.05) differences in richness among the four sampling sites (Figure 4C). The Gini–Simpson index was 0.85, 0.86, 0.76, and 0.49, respectively. For Puli Township, the endophytic fungal diversity was higher in August (summer) than in December (winter).

**Figure 5:**
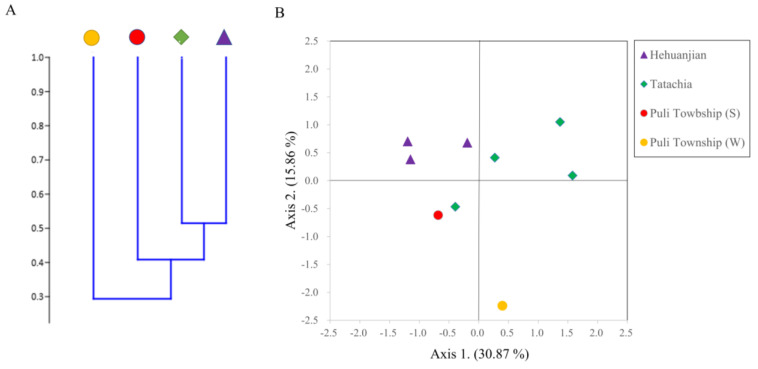
Bray–Curtis cluster tree (**A**) and principal component analysis (PCA) (**B**) showing results of beta diversity analysis of endophytic fungal isolates from *Cirsium kawakamii* in three sampling area samples (including two seasons in Puli Township; (S) = summer and (W) = winter).

The Bray–Curtis cluster tree constructed with PAST indicated that *C. kawakamii* endophytic fungal diversity clustered into two groups. Among these groups, the results from the two seasons in Puli Township were divided into two groups. (Figure 5A). The PCA results explained 46.73% of the total variation in the fungal community structure. Among the principal components, the first component was 30.88% and the second was 15.86%. For the first principal component, the communities of Hehuanjan were more concentrated than those of Tatachia. The second principal component distinguished different communities in Puli Township (winter) and others (Figure 5B).

### 3.4. Identification of Phytopathogen-like Endophytic Fusarium sp. Isolates

The morphology of the phytopathogen-like endophytic Fusarium sp. isolates were observed in PSA and PDA media, the colony floccose with abundant aerial mycelium, irregular margins, lobate, serrate, or filiform (Figure S1). Further, the colonies showed different colors in varying culture conditions. In PSA incubation at 20 °C with 12 h of light and dark, the colony surface showed white to pale vinaceous and reverse livid pink to pale violet, lacking diffusible pigment (Figure S1A,B). During incubation at 20 °C in the dark, the colony surface showed yellow to livid vinaceous and reverse deep vinaceous, lacking diffusible pigment (Figure S1C,D). In PDA incubation at 20 °C, the colony surface showed flesh to white and reverse pale luteous, lacking diffusible pigment (Figure S1E–H). In the PSA medium, the colonies contained short plump monophialides (conidiophores) (Figure S1I). The macroconidia were sickle-shaped, with a foot-shaped foot cell and a hooked apical cell, and 1–5 septa. Macroconidia with three septa accounted for 54.4% of the measured spores (Figure S1J). The microconidia were single-celled and club-shaped, with blunt ends (Figure S1K). The chlamydospores formed by the macroconidia or hyphae were round or elongated (Figure S1L,M). In addition, the colony appearance description and conidia size measurements of these isolates are recorded in Table S3. These morphological observations indicate that FTSC-like isolates in this study may be classified as section Sporotrichiella of *Fusarium* [42]. 

A BLASTn search of the EF-1α sequence of each isolate against *Fusarium* databases showed that all isolates were most similar to FTSC members, based on the significant homology values (>99%, unpublished data). To further determine the identity of all isolates, a phylogenetic analysis with the partial ITS and EF-1α sequences along with confirmed FTSC species was conducted. Two of these isolates (CKK 8020 and CKK 9035) were clustered with FTSC 21 (*Fusarium* sp.), and four isolates (CKK 3008, CKK 3019, CKK 4070, and CKK 9047) were similar to FTSC 4 (*F. avenaceum*). Three isolates (CKS 2002, CKS 2006, and CKS 2056) and four other isolates (CKK6059, CKS 5024, CKS 5039, and CKS 5099), respectively, formed two separate clades on the tree (Figure S2).

### 3.5. Pathogenicity Tests of Phytopathogen-like Endophytic Fusarium sp. Isolates on Apple, Potato, and Wheat

All 13 endophytic *Fusarium* spp. isolates belonging to the FTSC exhibited pathogenicity to potato, apple, and wheat. Among these isolates, CKK6059 showed the highest virulence on the plants. Fuji apples were inoculated with 2 μL of a CKK6059 spore suspension at 10^5^ conidia/mL and kept at 20 °C in an illuminated environment. At 7 d post-inoculation, the apples exhibited brown, circular, and slightly sunken lesions. Observation was continued until day 14, when the lesion diameter was 11.8 mm. At day 28 post-inoculation, the lesion diameter was 13.8 mm (Figure S3A,B). The apples were cut open and showed brown water-soaked lesions.

Potatoes inoculated with 20 μL of a CKK6059 spore suspension (10^5^ conidia/mL) were kept at 4 °C in the dark. At 28 d post-inoculation, no obvious symptoms were observed on the surface of the potatoes, which were indistinguishable from those of the control (Figure S3C), whereas their internal tissue was brown and dry (Figure S3D). The potatoes that were maintained at 20 °C for an additional 28 d showed their skins turning black (Figure S3E), with occasional sunken patches. The wound caused by the micropipette tip was covered with hyphae, and the internal tissues were hollowed out and necrotic, with a blackish-brown color. Hyphae were also observed in the hollowed-out areas (Figure S3F).

At 10 d post-inoculation, the wheat seedlings that were inoculated via wounding showed poor growth, root rot, and blackish-brown lesions, with white hyphae entwined at the base of the stem (Figure S3G,H). The leaves were dried, with irregular brown lesions (Figure S3I). In the non-wounding inoculation treatment group, wheat seedlings produced the same symptoms, while the disease progressed slower, with the symptoms taking >10 d to appear.

FTSC-like fungi were isolated from every diseased region of infested plants, indicating that these fungi were pathogens to potato, apple, and wheat.

## 4. Discussion

The endophytic fungal community of *C. kawakamii* endemic to Taiwan was characterized. Based on morphology and ITS nucleotide sequencing, 1814 endophytic fungal isolates within Ascomycota and Basidiomycota were identified. An additional 22 fungal isolates could not be identified. In Ascomycota, we identified 5 classes and 1 incertae sedis, 35 orders (with 1 incertae sedis), and 62 genera. In Basidiomycota, we identified two classes, four orders (including one incertae sedis), and five genera. The Shannon indices obtained here indicated that the endophytic fungal flora of *C. kawakamii* was highly diverse and rich. However, the genus accumulation curve based on the bootstrap estimator did not reach an asymptote; this suggests that, in this study, *C. kawakamii* had more endophytic fungal genera than the 68 that we identified. Furthermore, this suggests that the isolation methods used here may not have completely identified all of the fungi present. To estimate endophytic fungal diversity by culture methods may be underrated due to the fungal culture or isolation conditions [43,44]. For example, Arnold [45] indicated that more Basidiomycota genera were detected in *Pinus taeda* using molecular biological methods than were obtained using isolation methods. Therefore, this study will consider using molecular biology techniques and conducting more DNA sequence analyses in the future to improve the accuracy of the diversity analysis.

The dominant fungal genera isolated from the internal tissues of *C. kawakamii* were *Colletotrichum*, *Fusarium*, *Phomopsis*, and *Xylaria*. According to previous studies, the dominant fungal genera found in woody plants have included *Botryosphaeria*, *Cladosporium*, *Colletotrichum*, *Fusarium*, and *Xylaria* [7,18]. However, there has been limited research into the endophytic fungal communities of herbaceous plants. The endophytic fungal communities of herbaceous plants are less diverse and rich than those of woody plants. For example, for herbaceous plants, Gange et al. [46] found that the dominant endophytic fungi were *Acremonium*, *Alternaria*, *Cladosporium*, and *Epicoccum*. D’Amico et al. [20] reported that *Plectosporium* and *Fusarium* were the major endophytic fungal genera in lettuce, celery, and fennel tissue samples. Eschen et al. [27] analyzed the endophytic fungal community of *Cirsium arvense*, finding that the dominant genera were *Acremonium*, *Cladosporium*, *Phomopsis*, and *Trichothecium*. The endophytic fungal community composition of *C. kawakamii* exhibited high diversity and richness, being more similar to those previously reported for woody plants than for herbaceous plants. This difference may be attributed to environmental factors and to the plant community in which *C. kawakamii* occurs. Selim et al. [47] reported that indigenous crops that grew in biologically and ecologically diverse environments had highly diverse and rich endophytic fungal communities. As Taiwan is located in a subtropical zone with a warm and humid climate, the island is rich in flora and fauna. *Cirsium kawakamii* grows in subtropical, temperate, and cold zones with complex forest ecosystems and high species richness. Being endemic to Taiwan, *C. kawakamii* occupies a unique ecological niche, growing among various woody plants with which endophytic fungi have a long history of evolution and interaction.

The diversities of the endophytic fungal community of the *C. kawakamii* in two high-altitude sampling points (Tatachia and Hehuanjian) were not significantly different from that of the summer in Puli Township but were significantly higher than that of the winter in Puli Township. Endophytic fungal diversity was higher in tropical regions than in temperate and cold regions [19,48,49]. Tatachia and Hehuanjian tend to have a temperate climate, whereas Puli Township tends to have a subtropical climate. Nonetheless, based on our findings, Tatachia and Hehuanjian exhibited higher endophytic fungal diversity than Puli Township. This may be because Puli Township presents an artificial cultivation environment for *C. kawakamii*. Although it is an open area for cultivating multiple crops, it is less biodiverse than Tatachia and Hehuanjian. Plants growing in cultivated environments exhibit lower endophytic fungal diversity than those growing in primitive forests [50]. According to the beta diversity analysis in this study, Hehuanjian exhibited lower endophytic fungal community similarity between sampling points than Tatachia. Plant endophytic fungal communities are less similar in cold regions than those in temperate and tropical regions. Microorganisms are highly sensitive to the environment, and fungal community composition is easily influenced by environmental factors [19,51]. Hehuanjian typically has a cold climate, whereas Tatachia is typically temperate. The harsh and constantly changing environment of Hehuanjian makes it difficult for endophytic fungi to accumulate in plant tissues, and the scarcity of *C. kawakamii* in the area leads to high heterogeneity among the endophytic fungal communities at the different sampling points. This result also suggests that a plant’s endophytic fungal community can reflect its growth environment [52]. In addition, Arnold et al. [53] reported that endophytic fungal community similarity decreased as the distance between the plants increased. The *C. kawakamii* planted in Puli Township were transplanted there from the Tatachia area, and the endophytic fungal community in *C. kawakamii* in Puli Township may similarly have been imported from the Tatachia area, resulting in their higher endophytic community similarity. Endophytic fungi often move with the host to new areas (via co-introduction), and subsequent environmental influences may alter the fungal community [54]. The presence or absence of some endophytic fungi is therefore easily influenced by the environment [55,56]. In addition, during the sampling process, we observed that the population of *C. kawakamii* in the Hehuanjian area was limited, with only a few plants at each sampling site. Subsequent analysis revealed that the genus composition of endophytic fungi in *C. kawakamii* among the sampling sites in Hehuanjian was similar, but the population numbers varied significantly. This outcome differed from the analysis of Tatachia samples. Plant endophytic fungal communities are generally less similar in cold regions compared with temperate and tropical regions, as fungal community composition is easily influenced by environmental factors [19]. Hehuanjian typically experiences a cold climate, whereas Tatajia typically has a temperate climate. The harsh and constantly changing environment in Hehuanjian may make it challenging for endophytic fungi to accumulate in plant tissues. This result also suggests that a plant’s endophytic fungal community can be indicative of its growth environment.

To evaluate the effects of seasonal and geographical factors on the endophytic fungal community of *C. kawakamii*, we compared the endophytic fungal communities between the summer and winter samples from Puli Township and between the different regions. For Puli Township, endophytic fungal community similarity was lower between the seasons than between the different regions, based on the beta diversity analysis. This indicated that the endophytic fungal community of *C. kawakamii* in Puli Township was primarily affected by seasonal factors. The distribution and composition of endophytic fungal communities in the same plant were affected by seasonal and geographical factors. However, the primary factors affecting endophytic fungal communities varied between studies [57,58]. Mishra et al. [57] found that seasonal factors were more important than geographical factors in a study of the endophytic fungal community of *Tinospora cordifolia*. However, the results of an endophytic fungal community survey of *Quercus ilex* and *Laurus nobilis* revealed that geographical factors had a more notable effect than seasonal factors [56]. As mentioned above, seasonal and geographical factors can both affect endophytic fungal communities, although the main influencing factor may differ depending on the host plant species. For the plants from Puli Township, fungal community diversity was higher for the samples collected in summer than for those collected in winter. Seasonal differences can affect endophytic fungal communities, with temperature, humidity, and rainfall considered the main influencing factors [57,59]. Although endophytic fungal community diversity is lower in warmer environments than in cooler environments, the correlation with temperature is not observed for all endophytic fungi. For example, the abundance of the endophytic fungus *Ascochyta fagi* in Japanese beech decreases as temperature increases during plant growth [55]. Krishnamurthy et al. [59] pointed out that the appearance of endophytic fungi such as *Humicola fuscoatra*, *Botryosphaeria subglobosa*, *Torula herbarum*, and *Microsphaeropsis* sp. Endophytic fungi used in Chinese herbal medicines can only be isolated from plants during the wet season. The endophytic fungal communities are more diverse in plants grown in humid seasons than in those grown in dry seasons because the reproductive bodies of endophytic fungi can grow and form symbiotic relationships with plants under high humidity and suitable temperatures [60]. In Puli Township, during the sampling in August 2013, the temperature was 17.9–31.1 °C, with a relative humidity of 80%; for the sampling in December, the temperature was 6.0–25.3 °C, with a relative humidity of 75% (historical meteorological observation records of the Central Weather Bureau). Thus, the climate in Puli Township in December 2013 was cold and dry, which was not conducive to the accumulation of endophytic fungi in *C. kawakamii*, resulting in the lower endophytic fungal community diversity and richness during the winter cultivation of *C. kawakamii* in Puli Township. Although this is a preliminary finding, further long-term and multi-seasonal sampling will provide more useful information.

*Fusarium* is a common endophytic fungal genus in plants [47,56]. Our findings reveal that the several endophytic *Fusarium* isolates (potentially in the *F. tricinctum* species complex (FTSC) based on our morphological and molecular methods) are pathogenic in apples, potatoes, and wheat. This confirms that some endophytic fungi found in the leaves of *C. kawakamii* are potentially pathogenic toward crops. Pathogenic *Fusarium* species can also establish symbiotic relationships with plants as endophytes [61]. For example, *F. circinatum*, the causative agent of pine pitch canker, is an endophyte in grass tissues [62]. Endophytic fungi evolve from fungal pathogens, leading to the conversion of some endophytic fungi between endophytes and fungal pathogens [54,63]. Moreover, fungal endophytes have various types of symbiotic relationships with plants [64,65]. Several phytopathogens can latently infect hosts or nonhosts [66,67]. For example, *Verticillium dahliae*, a phytopathogen that infects more than 400 plant species, is also found as an endophytic fungus in several plants [68]. Therefore, confirming that endophytic fungi have pathogenic potential is important. Primary forests are successional environments in which pathogens play an important role, and many pathogens produce large numbers of propagules in host debris and release them into the environment [69]. Pathogens coexisting within the same ecological niche as *C. kawakamii* possibly establish a prolonged symbiotic association with this plant, evolving into endophytes over time. *Fusarium circinatum*, a pine pathogen, can be a symptomless endophyte to some grasses, such as *Briza maxima* or *Ehrharta erecta*, and can stabilize its population and transmission [62]. *Cirsium kawakamii* may be similar to these grasses in terms of its ecological status; it has evolved to become a fungal inoculum reservoir for various fungi in the local environment, enabling a variety of endophytic as well as pathogenic fungi to accumulate in tissues.

## 5. Conclusions

This study analyzed the endophytic fungal diversity of the indigenous herb *Cirsium kawakamii* in Taiwan. It identified a diverse fungal community with dominant genera, including *Colletotrichum*, *Fusarium*, *Phomopsis*, and *Xylaria*. Seasonal and geographic variations were observed, and some endophytic *Fusarium* isolates belonging to the *Fusarium tricinctum* species complex (FTSC) played the role as plant pathogen to apple, potato, or wheat. This research shed light on the unique fungal diversity associated with *C. kawakamii* and its potential ecological implications. 

At present, there is likely no immediate risk of large-scale crop disease outbreaks associated with *C. kawakamii*. However, as *C. kawakamii* is used in Chinese herbal medicine and may be cultivated in various locations in the future, it is essential to establish a monitoring system to track the dynamics of endophytic pathogenic fungi in the environment and mitigate the risk of their spreading to agricultural crop cultivation regions.

## Figures and Tables

**Table 1:** Endophytic fungi of *Cirsium kawakamii* and their isolation frequencies for the three study sites: Puli Township, Hehuanjian, and Tatajia.

Fungal Family	Fungal Genus	Number of Isolates (Isolation Frequency (%))							
		Hehuanjian		Tatachia		Puli Township		Total	
Ascomycota									
Dothideomycetes									
Cladosporiaceae	*Cladosporium*	1	(0.3)	4	(0.4)	16	(3.3)	21	(1.2)
	*Toxicocladosporium*	0	(0.0)	1	(0.1)	0	(0.0)	1	(0.1)
Coniothyriaceae	*Coniothyrium*	2	(0.5)	0	(0.0)	0	(0.0)	2	(0.1)
Corynesporascaceae	*Corynespora*	0	(0.0)	4	(0.4)	26	(5.4)	30	(1.7)
Didymellaceae	*Leptosphaerulina*	0	(0.0)	11	(1.2)	0	(0.0)	11	(0.6)
	*Phoma*	57	(14.9)	14	(1.5)	2	(0.4)	73	(4.0)
Didymosphaeriaceae	*Paraconiothyrium*	0	(0.0)	1	(0.1)	0	(0.0)	1	(0.1)
Massarinaceae	*Saccharicola*	4	(1.0)	0	(0.0)	0	(0.0)	4	(0.2)
Mycosphaerellaceae	*Cercospora*	3	(0.8)	2	(0.2)	32	(6.7)	37	(2.0)
	*Mycocentrospora*	3	(0.8)	0	(0.0)	0	(0.0)	3	(0.2)
	*Septoria*	1	(0.3)	1	(0.1)	0	(0.0)	2	(0.1)
Phaeosphaeriaceae	*Paraphoma*	15	(3.9)	25	(2.6)	1	(0.2)	41	(2.3)
	*Phaeosphaeria*	2	(0.5)	2	(0.2)	0	(0.0)	4	(0.2)
Pleosporaceae	*Alternaria*	8	(2.1)	6	(0.6)	5	(1.0)	19	(1.0)
	*Bipolaris*	0	(0.0)	0	(0.0)	5	(1.0)	5	(0.3)
	*Curvularia*	1	(0.3)	0	(0.0)	1	(0.2)	2	(0.1)
Botryosphaeriaceae	*Botryosphaeria*	0	(0.0)	1	(0.1)	1	(0.2)	2	(0.1)
	*Neofusicoccum*	0	(0.0)	2	(0.2)	0	(0.0)	2	(0.1)
Phyllostictaceae	*Phyllosticta*	0	(0.0)	0	(0.0)	5	(1.0)	5	(0.3)
Eurotiomycetes									
Aspergillaceae	*Aspergillus*	8	(2.1)	0	(0.0)	8	(1.7)	16	(0.9)
	*Penicillium*	0	(0.0)	1	(0.1)	12	(2.5)	13	(0.7)
Leotiomycetes									
Dermateaceae	*Neofabraea*	19	(5.0)	3	(0.3)	0	(0.0)	22	(1.2)
Incertae sedis	*Scytalidium*	1	(0.3)	0	(0.0)	0	(0.0)	1	(0.1)
Pseudeurotiaceae	*Geomyces*	0	(0.0)	0	(0.0)	1	(0.2)	1	(0.1)
Rhytismataceae	*Leptostroma*	0	(0.0)	2	(0.2)	0	(0.0)	2	(0.1)
Pezizomycetes									
Morchellaceae	*Morchella*	0	(0.0)	1	(0.1)	0	(0.0)	1	(0.1)
Sordariomycetes									
Amphisphaeriaceae	*Discostroma*	0	(0.0)	2	(0.2)	0	(0.0)	2	(0.1)
	*Pestalotiopsis*	0	(0.0)	0	(0.0)	1	(0.2)	1	(0.1)
Apiosporaceae	*Arthrinium*	1	(0.3)	3	(0.3)	0	(0.0)	4	(0.2)
Bionectriaceae	*Bionectria*	1	(0.3)	1	(0.1)	0	(0.0)	2	(0.1)
Chaetomiaceae	*Chaetomium*	0	(0.0)	1	(0.1)	17	(3.5)	18	(1.0)
Diatrypaceae	*Eutypa*	0	(0.0)	0	(0.0)	2	(0.4)	2	(0.1)
	*Incertae sedis*	0	(0.0)	0	(0.0)	1	(0.2)	1	(0.1)
Glomerellaceae	*Colletotrichum*	70	(18.3)	310	(32.6)	164	(34.2)	544	(30.0)
Gnomoniaceae	*Ophiognomonia*	0	(0.0)	3	(0.3)	0	(0.0)	3	(0.2)
Hypocreaceae	*Trichoderma*	3	(0.8)	0	(0.0)	0	(0.0)	3	(0.2)
Incertae sedis	*Hansfordia*	0	(0.0)	2	(0.2)	0	(0.0)	2	(0.1)
	*Incertae sedis*	0	(0.0)	2	(0.2)	0	(0.0)	2	(0.1)
	*Myrothecium*	0	(0.0)	1	(0.1)	10	(2.1)	11	(0.6)
Lasiosphaeriaceae	*Apodus*	0	(0.0)	1	(0.1)	1	(0.2)	2	(0.1)
	*Cercophora*	33	(8.6)	0	(0.0)	0	(0.0)	33	(1.8)
	*Podospora*	9	(2.3)	0	(0.0)	0	(0.0)	9	(0.5)
Leptosphaeriaceae	*Leptosphaeria*	14	(3.7)	11	(1.2)	0	(0.0)	25	(1.4)
Magnaporthaceae	*Gaeumannomyces*	1	(0.3)	3	(0.3)	0	(0.0)	4	(0.2)
	*Ophioceras*	0	(0.0)	4	(0.4)	0	(0.0)	4	(0.2)
Nectriaceae	*Fusarium*	73	(19.1)	120	(12.6)	10	(2.1)	203	(11.2)
	*Ilyonectria*	0	(0.0)	1	(0.1)	0	(0.0)	1	(0.1)
Plectosphaerellaceae	*Acremonium*	0	(0.0)	12	(1.3)	3	(0.6)	15	(0.8)
	*Acrostalagmus*	0	(0.0)	0	(0.0)	1	(0.2)	1	(0.1)
	*Plectosphaerella*	1	(0.3)	10	(1.1)	31	(6.5)	42	(2.3)
Trichosphaeriaceae	*Nigrospora*	0	(0.0)	6	(0.6)	6	(1.3)	12	(0.7)
Valsaceae	*Incertae sedis*	0	(0.0)	1	(0.1)	0	(0.0)	1	(0.1)
	*Phomopsis*	23	(6.0)	56	(5.9)	21	(4.4)	100	(5.5)
Xylariaceae	*Annulohypoxylon*	1	(0.3)	1	(0.1)	4	(0.8)	6	(0.3)
	*Biscogniauxia*	0	(0.0)	1	(0.1)	1	(0.2)	2	(0.1)
	*Daldinia*	0	(0.0)	3	(0.3)	10	(2.1)	13	(0.7)
	*Hypoxylon*	4	(1.0)	10	(1.1)	25	(5.2)	39	(2.1)
	*Incertae sedis*	0	(0.0)	0	(0.0)	1	(0.2)	1	(0.1)
	*Nemania*	1	(0.3)	35	(3.7)	6	(1.3)	42	(2.3)
	*Nodulisporium*	0	(0.0)	6	(0.6)	4	(0.8)	10	(0.6)
	*Xylaria*	18	(4.7)	260	(27.3)	43	(9.0)	321	(17.7)
Incertae sedis									
Incertae sedis	*Trichocladium*	1	(0.3)	0	(0.0)	0	(0.0)	1	(0.1)
Basidiomycota									
Agaricomycetes									
Ceratobasidiaceae	*Ceratobasidium*	2	(0.5)	4	(0.4)	0	(0.0)	6	(0.3)
	*Rhizoctonia*	1	(0.3)	0	(0.0)	0	(0.0)	1	(0.1)
*Incertae sedis*	*Rigidoporus*	0	(0.0)	0	(0.0)	1	(0.2)	1	(0.1)
Psathyrellaceae	*Coprinellus*	1	(0.3)	0	(0.0)	1	(0.2)	2	(0.1)
Ustilaginomycetes									
Ustilaginaceae	*Pseudozyma*	0	(0.0)	0	(0.0)	1	(0.2)	1	(0.1)
Unknown		3		11		8		22	
Total		386		962		488		1836	

## Data Availability

The raw data that support analysis of this study are not openly available due to reasons of sensitivity, and are available from the corresponding authors upon reasonable request.

## Supplementary Materials

The following supporting information can be downloaded at: https://www.mdpi.com/article/10.3390/jof9111076/s1, Figure S1: Effects of different culture media on phytopathogen-like endophytic *Fusarium* sp. isolates in this study. For colony morphology: colony morphology of *Fusarium* sp. isolates cultured on potato sucrose agar (PSA) with 12 h light and dark (A,B), colony morphology of *Fusarium* sp. isolates cultured on PSA in the dark (C,D), colony morphology of *Fusarium* sp. isolates cultured on potato dextrose agar (PDA) with 12 h light and dark (E,F), colony morphology of *Fusarium* sp. isolates cultured on PDA in the dark (G,H), (I–M) conidial morphology of *Fusarium* sp. isolates, (I) short and plump monophialide conidiophore, (J) macroconidia, (K) microconidia, (L) chlamydospore, (M) chlamydospore. Bar, 10 μm. Figure S2: A phylogenetic tree was constructed using the maximum likelihood (ML) method, based on combined sequences of the internal transcribed spacer (ITS) sequence and translation elongation factor 1 alpha (EF-1α) gene of endophytic *Fusarium* isolates from *Cirsium kawakamii* in this study (●), related *Fusarium tricinctum* species complex (FTSC) isolates (▓), and *Fusarium fujikuroi* species complex (FFSC) isolates (◆) available at NCBI. The tree was rooted with *Fusarium solani* strain NRRL 46643 (▲) as the outgroup, and bootstrap values were determined using 1000 replicates. The scale bar indicates the nucleotide changes. Figure S3: Symptoms of potato, apple, and wheat inoculated with *Fusarium* sp. CKK6059 spore suspension (10^6^ conidia/mL). (A,B) Symptoms on apple ‘Fuji’; (C,D) potato inoculated with CKK6059, after 28 d of incubation at 4 °C; (E,F) potato inoculated with CKK6059, after 28 d of incubation at 20 °C. For wheat: growth inhibition (G), necrosis and root-rot of wheat (H), and leaf blight of wheat (I). Control (CK): inoculation with sterile soil extract broth. (white bar = 1 cm) Table S1: Endophytic *Fusarium tricinctum* species complex (FTSC)-like isolates collected from *Cirsium kawakamii* in this study. Table S2: GenBank strain numbers for phylogenetic tree analysis of the internal transcribed spacer (ITS) and translation elongation factor subunit alpha (EF-1α) sequences in this study. Table S3: Morphological characteristics of the endophytic *Fusarium tricinctum* species complex (FTSC)-like isolates collected from *Cirsium kawakamii* in this study [70,71].

## References

[1] Petrini O., Andrews J., Hirano S. (1991). Fungal endophytes of tree leaves. Microbial Ecology of Leaves.

[2] Wilson D. (1995). Endophyte: The evolution of a term, and clarification of its use and definition. Oikos.

[3] Stone J.K., Bacon C.W., White J.F., Bacon C.W., White J.F. (2000). An overview of endophytic microbes: Endophytism defined. Microbial Endophytes.

[4] Nair D.N., Padmavathy S. (2014). Impact of endophytic microorganisms on plants, environment and humans. Sci. World J..

[5] Krings M., Taylor T.N., Hass H., Kerp H., Dotzler N., Hermsen E.J. (2007). Fungal endophytes in a 400-million-yr-old land plant: Infection pathways, spatial distribution, and host responses. New Phytol..

[6] Brundrett M.C., Schulz B.J.E., Boyle C.J.C., Sieber T.N. (2006). Understanding the roles of multifunctional mycorrhizal and endophytic fungi. Microbial Root Endophytes.

[7] Rodriguez R.J., White J.F., Arnold A.E., Redman R.S. (2009). Fungal endophytes: Diversity and functional roles. New Phytol..

[8] Hartley S.E., Gange A.C. (2009). Impacts of plant symbiotic fungi on insect herbivores: Mutualism in a multitrophic context. Ann. Rev. Entomol..

[9] Kharwar R.N., Mishra A., Gond S.K., Stierle A., Stierle D. (2011). Anticancer compounds derived from fungal endophytes: Their importance and future challenges. Nat. Prod. Rep..

[10] Preethi K., Manon Mani V., Lavanya N., Patil R.H., Maheshwari V.L. (2021). Endophytic Fungi: A potential source of bioactive compounds for commercial and therapeutic applications. Endophytes.

[11] Zhao J., Shan T., Mou Y., Zhou L. (2011). Plant-derived bioactive compounds produced by endophytic fungi. Mini. Rev. Med. Chem..

[12] Stierle A., Strobel G., Stierle D. (1993). Taxol and taxane production by *Taxomyces andreanae*, an endophytic fungus of pacific yew. Science.

[13] Chakravarthi B.V.S.K., Das P., Surendranath K., Karande A.A., Jayabaskaran C. (2008). Production of paclitaxel by *Fusarium solani* isolated from *Taxuscelebica*. J. Biosci..

[14] Pongcharoen W., Rukachaisirikul V., Phongpaichit S., Kuehn T., Pelzing M., Sakayaroj J., Taylor W.C. (2008). Metabolites from the endophytic fungus *Xylaria* sp. PSU-D14. Phytochemistry.

[15] Strobel G., Daisy B. (2003). Bioprospecting for microbial endophytes and their natural products. Microbiol. Mol. Biol. Rev..

[16] Leuchtmann A. (1992). Systematics, distribution, and host specificity of grass endophytes. Nat. Toxins.

[17] Hoffman M.T., Arnold A.E. (2008). Geographic locality and host identity shape fungal endophyte communities in cupressaceous trees. Mycol. Res..

[18] Arnold A.E., Henk D.A., Eells R.L., Lutzoni F., Vilgalys R. (2007). Diversity and phylogenetic affinities of foliar fungal endophytes in loblolly pine inferred by culturing and environmental PCR. Mycologia.

[19] Arnold A.E., Lutzoni F. (2007). Diversity and host range of foliar fungal endophytes: Are tropical leaves biodiversity hotspots?. Ecology.

[20] D’Amico M., Frisullo S., Cirulli M. (2008). Endophytic fungi occurring in fennel, lettuce, chicory, and celery-commercial crops in southern Italy. Mycol. Res..

[21] Gazis R., Chaverri P. (2010). Diversity of fungal endophytes in leaves and stems of wild rubber trees (*Hevea brasiliensis*) in Peru. Fungal Ecol..

[22] MacArthur D., McGee P. (2000). A comparison of the endophytic fungi from leaves of *Banksia integrifolia* at three sites on the east coast of Australia. Australas. Mycol..

[23] Vega F.E., Simpkins A., Aime M.C., Posada F., Peterson S.W., Rehner S.A., Infante F., Castillo A., Arnold A.E. (2010). Fungal endophyte diversity in coffee plants from Colombia, Hawai’i, Mexico and Puerto Rico. Fungal Ecol..

[24] Peng C.I., Boufford D.E., Hsieh C.F., Kuo C.S., Ohashi H., Su H.J. (2003). Cirsium. Flora of Taiwan.

[25] Lu Y.S. (2011). Investigation of antioxidative components in *Cirsium kawakamii* Hayata. Master’s Thesis.

[26] Zhao Z.W., Chang J.C., Lin L.W., Tsai F.H., Chang H.C., Wu C.R. (2018). Comparison of the hepatoprotective effects of four endemic *Cirsium* species extracts from Taiwan on ccl4-induced acute liver damage in C57BL/6 Mic. Int. J. Mol. Sci..

[27] Eschen R., Hunt S., Mykura C., Gange A.C., Sutton B.C. (2010). The foliar endophytic fungal community composition in *Cirsium arvense* is affected by mycorrhizal colonization and soil nutrient content. Fungal Biol..

[28] Bills G.F., Redlin S., Carris L., Redlin S., Carris L. (1996). Isolation and analysis of endophytic fungal communities from woody plants. Endophytic Fungi in Grasses and Woody Plants: Systematics, Ecology, and Evolution.

[29] Goodwin D., Lee S. (1993). Microwave miniprep of total genomic DNA from fungi, plants, protists and animals for PCR. Biotechniques.

[30] White T.J., Bruns T.D., Lee S.B., Taylor J.W., Innis M.A., Gelfand D.H., Sninsky J.J., White T.J. (1990). Amplification and Direct Sequencing of Fungal Ribosomal RNA Genes for Phylogenetics. PCR Protocols: A Guide to Methods and Applications.

[31] Samuels G.J., Ismaiel A. (2011). Hypocrea peltata: A mycological Dr Jekyll and Mr Hyde?. Mycologia.

[32] O’Donnell K., Kistler H.C., Cigelnik E., Ploetz R.C. (1998). Multiple evolutionary origins of the fungus causing Panama disease of banana: Concordant evidence from nuclear and mitochondrial gene genealogies. Proc. Natl. Acad. Sci. USA.

[33] Thompson J.D., Higgins D.G., Gibson T.J. (1994). CLUSTAL W: Improving the sensitivity of progressive multiple sequence alignment through sequence weighting, position-specific gap penalties and weight matrix choice. Nucleic Acids Res..

[34] Kumar S., Stecher G., Li M., Knyaz C., Tamura K. (2018). MEGA X: Molecular Evolutionary Genetics Analysis across computing platforms. Mol. Biol. Evol..

[35] Laraba I., Busman M., Geiser D.M., O’Donnell K. (2022). Phylogenetic diversity and mycotoxin potential of emergent phytopathogens within the *Fusarium tricinctum* species complex. Phytopathology.

[36] Li F., Jiang X., Sun M., Xu H.L., Shi L., Shan W., Feng Y. (2014). Screening of culture conditions for pathogens of potato dry rot. Acta Agric. Scand.-B Soil Plant Sci..

[37] Bottalico A., Perrone G. (2002). Toxigenic *Fusarium* species and mycotoxins associated with head blight in small-grain cereals in Europe. Eur. J. Plant Pathol..

[38] Gao L.L., Zhang Q., Sun X.Y., Jiang L., Zhang R., Sun G.Y., Zha Y.L., Biggs A.R. (2013). Etiology of moldy core, core browning, and core rot of Fuji apple in China. Plant Dis..

[39] Gachango E., Hanson L., Rojas A., Hao J., Kirk W. (2012). *Fusarium* spp. causing dry rot of seed potato tubers in Michigan and their sensitivity to fungicides. Plant Dis..

[40] Wu A.B., Li H.P., Zhao C.S., Liao Y.C. (2005). Comparative pathogenicity of *Fusarium graminearum* isolates from China revealed by wheat coleoptile and floret inoculations. Mycopathologia.

[41] Sever Z., Ivic D., Kos T., Milicevic T. (2012). Identification of *Fusarium* species isolated from stored apple fruit in Croatia. Arh. Hig. Rada. Toksikol..

[42] Booth C. (1971). The Genus Fusarium.

[43] Araújo W.L., Maccheroni W., Aguilar-Vildoso C.I., Barroso P.A., Saridakis H.O., Azevedo J.L. (2001). Variability and interactions between endophytic bacteria and fungi isolated from leaf tissues of citrus rootstocks. Can. J. Microbiol..

[44] Hyde K., Soytong K. (2008). The fungal endophyte dilemma. Fungal Div..

[45] Arnold A.E. (2007). Understanding the diversity of foliar endophytic fungi: Progress, challenges, and frontiers. Fungal Biol. Rev..

[46] Gange A.C., Dey S., Currie A.F., Sutton B.C. (2007). Site-and species-specific differences in endophyte occurrence in two herbaceous plants. J. Ecol..

[47] Selim K., El-Beih A., Abdel-Rahman T., El-Diwany A. (2012). Biology of endophytic fungi. Curr. Res. Environ. Appl. Mycol..

[48] Arnold A., Maynard Z., Gilbert G., Coley P., Kursar T. (2000). Are tropical fungal endophytes hyperdiverse?. Ecol. Lett..

[49] Fröhlich J., Hyde K.D. (1999). Biodiversity of palm fungi in the tropics: Are global fungal diversity estimates realistic?. Biodivers. Conserv..

[50] Wu S.Y., Chen Y.J., Chung W.C., Chung W.H. (2017). Diversity of fungal endophytes in *Cinnamomum kanehirae* in Taiwan and control assessment on crop diseases. J. Plant Med..

[51] Higgins K.L., Arnold A.E., Miadlikowska J., Sarvate S.D., Lutzoni F. (2007). Phylogenetic relationships, host affinity, and geographic structure of boreal and arctic endophytes from three major plant lineages. Mol. Phylogenet. Evol..

[52] Zimmerman N.B., Vitousek P.M. (2012). Fungal endophyte communities reflect environmental structuring across a Hawaiian landscape. Proc. Natl. Acad. Sci. USA.

[53] Arnold A.E., Mejia L.C., Kyllo D., Rojas E.I., Maynard Z., Robbins N., Herre E.A. (2003). Fungal endophytes limit pathogen damage in a tropical tree. Proc. Natl. Acad. Sci. USA.

[54] Shipunov A., Newcombe G., Raghavendra A.K., Anderson C.L. (2008). Hidden diversity of endophytic fungi in an invasive plant. Am. J. Bot..

[55] Hashizume Y., Fukuda K., Sahashi N. (2010). Effects of summer temperature on fungal endophyte assemblages in Japanese beech (*Fagus crenata*) leaves in pure beech stands. Botany.

[56] Naik B.S., Shashikala J., Krishnamurthy Y. (2008). Diversity of fungal endophytes in shrubby medicinal plants of Malnad region, Western Ghats, Southern India. Fungal Ecol..

[57] Mishra A., Gond S.K., Kumar A., Sharma V.K., Verma S.K., Kharwar R.N., Sieber T.N. (2012). Season and tissue type affect fungal endophyte communities of the Indian medicinal plant *Tinospora cordifolia* more strongly than geographic location. Microb. Ecol..

[58] Göre M., Bucak C. (2007). Geographical and seasonal influences on the distribution of fungal endophytes in *Laurus nobilis*. For. Pathol..

[59] Krishnamurthy Y.L., Naik S.B., Jayaram S. (2008). Fungal communities in herbaceous medicinal plants from the Malnad region, Southern India. Microbes Environ..

[60] Naik B.S., Krishnappa M., Krishnamurthy Y. (2014). Biodiversity of endophytic fungi from seven herbaceous medicinal plants of Malnad region, Western Ghats, southern India. J. For. Res..

[61] Ma L.J., Geiser D.M., Proctor R.H., Rooney A.P., O’Donnell K., Trail F., Gardiner D.M., Manners J.M., Kazan K. (2013). *Fusarium* *pathogenomics*. Annu. Rev. Microbiol..

[62] Swett C.L., Porter B., Fourie G., Steenkamp E.T., Gordon T.R., Wingfield M.J. (2014). Association of the pitch canker pathogen *Fusarium circinatum* with grass hosts in commercial pine production areas of South Africa. South. For..

[63] Promputtha I., Lumyong S., Dhanasekaran V., McKenzie E.H.C., Hyde K.D., Jeewon R. (2007). A phylogenetic evaluation of whether endophytes become saprotrophs at host senescence. Microb. Ecol..

[64] Redman R.S., Dunigan D.D., Rodriguez R.J. (2001). Fungal symbiosis from mutualism to parasitism: Who controls the outcome, host or invader?. New Phytol..

[65] Stergiopoulos I., Gordon T.R. (2014). Cryptic fungal infections: The hidden agenda of plant pathogens. Front. Plant Sci..

[66] Chen Y.J., Lin Y.S., Pan H.R., Chung W.H. (2020). Distribution and multiplication of *Ralstonia solanacearum* strain race 1 biovar 4 in vegetable sweet potato cuttings. J. Phytopathol..

[67] Swanson J.K., Yao J., Tans-Kersten J., Allen C. (2005). Behavior of *Ralstonia solanacearum* race 3 biovar 2 during latent and active infection of geranium. Phytopathology.

[68] Malcolm G.M., Kuldau G.A., Gugino B.K., Jiménez-Gasco M. (2013). Hidden host plant associations of soilborne fungal pathogens: An ecological perspective. Phytopathology.

[69] Gilbert G.S. (2002). Evolutionary ecology of plant diseases in natural ecosystems. Annu. Rev. Phytopathol..

[70] Bennett R., Davis R.M. (2013). Method for rapid production of *Fusarium oxysporum* f. sp. vasinfectum chlamydospores. J. Cotton Sci..

[71] Seifert K.A., Cereal E., Cereal E., Cereal E. (1996). Fuskey: Fusarium Interactive Key.

