# Supplementary material for: Endophytic Fungal Diversity in Cirsium kawakamii from Taiwan
Source: J Fungi (Basel). 2023 Nov 3;9(11):1076. doi: 10.3390/jof9111076 (PMC10671896; doi:10.3390/jof9111076)
Supplement: Supplementary file 1 [file jof-09-01076-s001.zip › jof-2596654 Supplementary.pdf]

Supplementary information

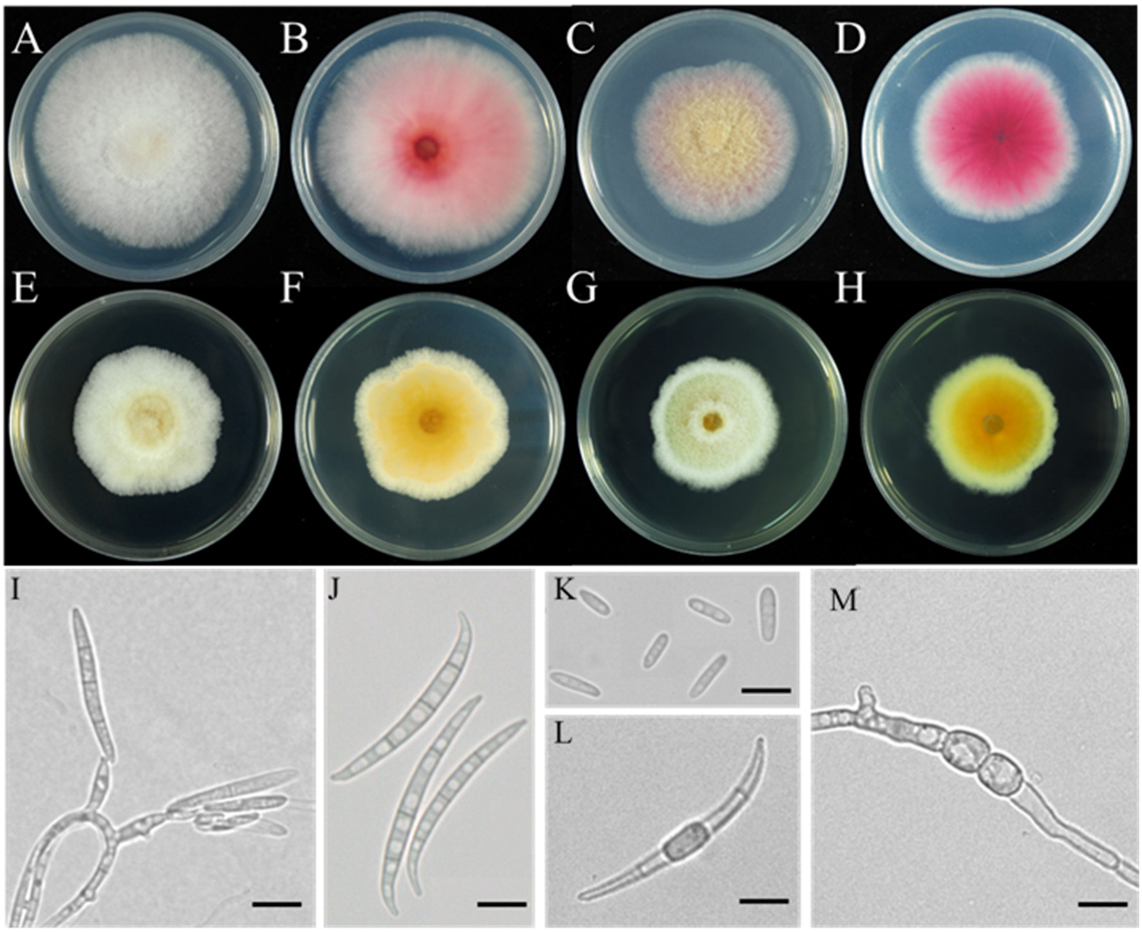

Figure S1. Effects of different culture media on phytopathogen-like endophytic *Fusarium* sp. isolates in this study. For colony morphology: Colony morphology of *Fusarium* sp. isolates cultured on potato sucrose agar (PSA) with 12 h light and dark (A and B), Colony morphology of *Fusarium* sp. isolates cultured on PSA in the dark (C and D), Colony morphology of *Fusarium* sp. isolates cultured on potato dextrose agar (PDA) with 12 h light and dark (E and F), Colony morphology of *Fusarium* sp. isolates cultured on PDA in the dark (G and H); I–M: Conidial morphology of *Fusarium* sp. isolates; I: Short and plump monophialide conidiophore; J: Macroconidia; K: Microconidia; L: Chlamydospore; M: Chlamydospore. Bar, 10 μm.

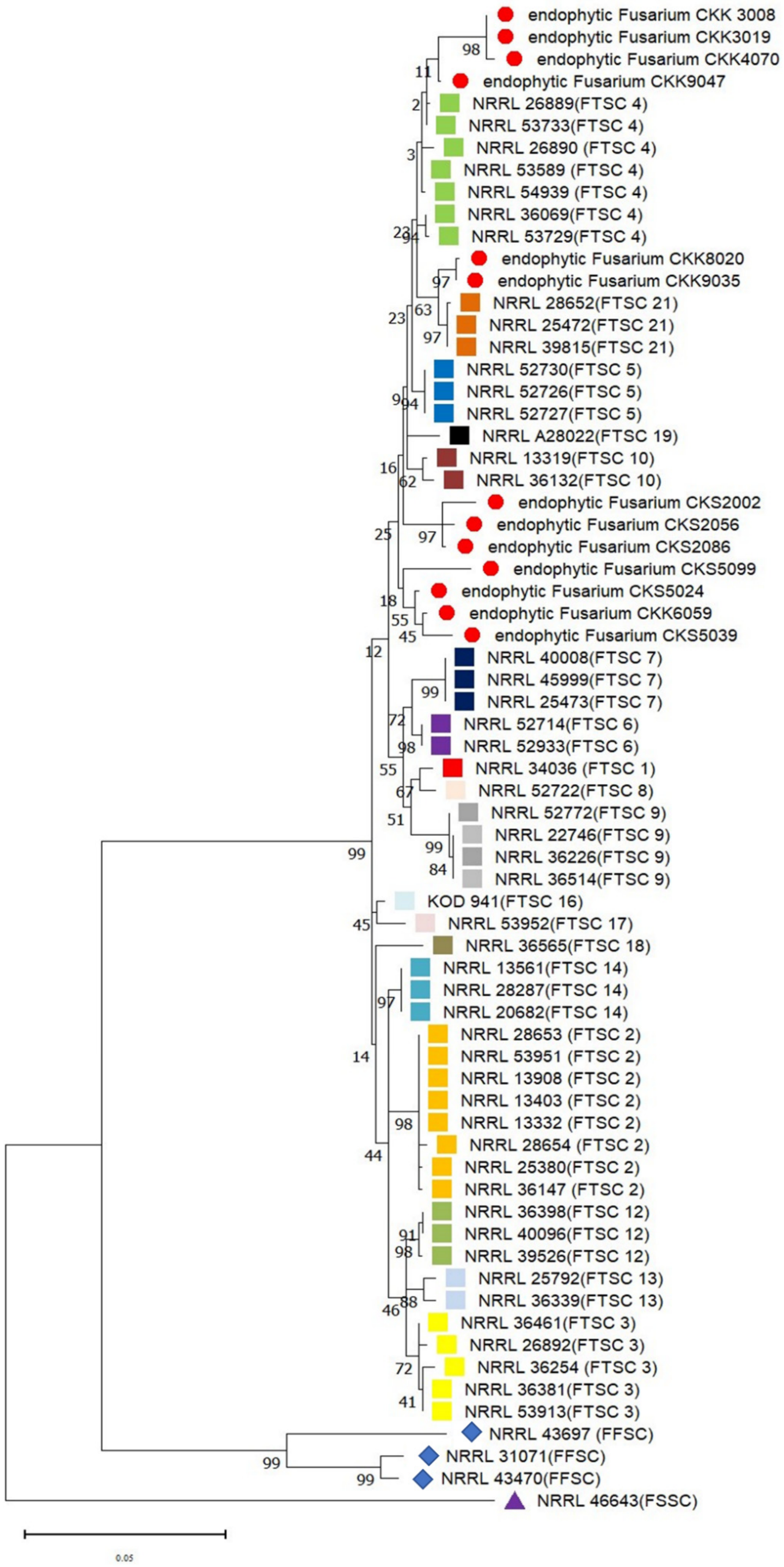
endophytic Fusarium CKK 3008
endophytic Fusarium CKK3019
endophytic Fusarium CKK4070
endophytic Fusarium CKK9047
NRRL 26889(FTSC 4)
NRRL 53733(FTSC 4)
NRRL 26890 (FTSC 4)
NRRL 53589 (FTSC 4)
NRRL 54939 (FTSC 4)
NRRL 36069(FTSC 4)
NRRL 53729(FTSC 4)
endophytic Fusarium CKK8020
endophytic Fusarium CKK9035
NRRL 28652(FTSC 21)
NRRL 25472(FTSC 21)
NRRL 39815(FTSC 21)
NRRL 52730(FTSC 5)
NRRL 52726(FTSC 5)
NRRL 52727(FTSC 5)
NRRL A28022(FTSC 19)
NRRL 13319(FTSC 10)
NRRL 36132(FTSC 10)
endophytic Fusarium CKS2002
endophytic Fusarium CKS2056
endophytic Fusarium CKS2086
endophytic Fusarium CKS5099
endophytic Fusarium CKS5024
endophytic Fusarium CKK6059
endophytic Fusarium CKS5039
NRRL 40008(FTSC 7)
NRRL 45999(FTSC 7)
NRRL 25473(FTSC 7)
NRRL 52714(FTSC 6)
NRRL 52933(FTSC 6)
NRRL 34036 (FTSC 1)
NRRL 52722(FTSC 8)
NRRL 52772(FTSC 9)
NRRL 22746(FTSC 9)
NRRL 36226(FTSC 9)
NRRL 36514(FTSC 9)
KOD 941(FTSC 16)
NRRL 53952(FTSC 17)
NRRL 36565(FTSC 18)
NRRL 13561(FTSC 14)
NRRL 28287(FTSC 14)
NRRL 20682(FTSC 14)
NRRL 28653 (FTSC 2)
NRRL 53951 (FTSC 2)
NRRL 13908 (FTSC 2)
NRRL 13403 (FTSC 2)
NRRL 13332 (FTSC 2)
NRRL 28654 (FTSC 2)
NRRL 25380(FTSC 2)
NRRL 36147 (FTSC 2)
NRRL 36398(FTSC 12)
NRRL 40096(FTSC 12)
NRRL 39526(FTSC 12)
NRRL 25792(FTSC 13)
NRRL 36339(FTSC 13)
NRRL 36461(FTSC 3)
NRRL 26892(FTSC 3)
NRRL 36254 (FTSC 3)
NRRL 36381(FTSC 3)
NRRL 53913(FTSC 3)
NRRL 43697 (FFSC)
NRRL 31071(FFSC)
NRRL 43470(FFSC)
NRRL 46643(FSSC)
0.05

Figure S2. A phylogenetic tree was constructed using the maximum likelihood (ML) method, based on combined sequences of the internal transcribed spacer (ITS) sequence and translation elongation factor 1 alpha (EF-1α) gene of endophytic *Fusarium* isolates from *Cirsium kawakamii* in this study (●), related *Fusarium tricinctum* species complex (FTSC) isolates (▓), and *Fusarium fujikuroi* species complex (FFSC) isolates (◆) available at NCBI. The tree was rooted with *Fusarium solani* strain NRRL 46643 (▲) as the outgroup, and bootstrap values were determined using 1000 replicates. The scale bar indicates the nucleotide changes.

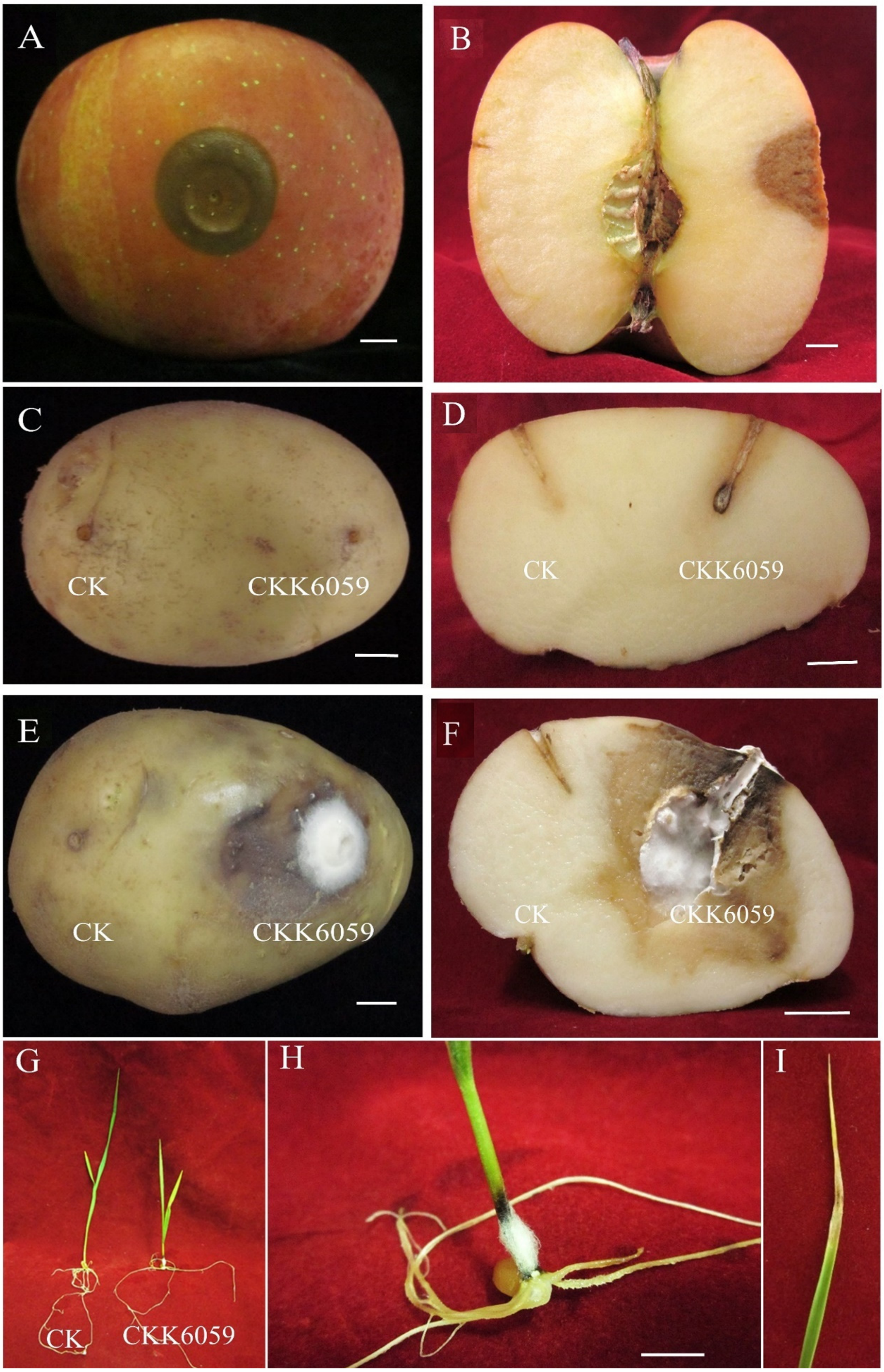
A
B
C
CK
CKK6059
D
CK
CKK6059
E
CK
CKK6059
F
CK
CKK6059
G
CK
CKK6059
H
I

Figure S3. Symptoms of potato, apple, and wheat inoculated with *Fusarium* sp. CKK6059 spore suspension (10^6 conidia/mL). A, B: Symptoms on apple ‘Fuji’; C, D: Potato inoculated with CKK6059, after 28 d of incubation at 4 °C; E, F: Potato inoculated with CKK6059, after 28 d of incubation at 20 °C. For wheat: Growth inhibition (G), Necrosis and root-rot of wheat (H), and Leaf blight of wheat (I). Control (CK): inoculation with sterile soil extract broth. (white bar = 1 cm)

Table S1. Endophytic *Fusarium tricinctum* species complex (FTSC)-like isolates collected from *Cirsium kawakamii* in this study

Collection area[1]	Collection site	Coordinates	Elevation (m)	Collection date	Fungal isolates
Tatachia	1	23°31'15.2"N 120°53'41.7"E	2,474	2013/10/11	CKK 3008、CKK 3019、CKK 4070
	2	23°28'29.1"N, 120°52'21.1"E	2,462	2013/10/11	CKK 6059
	3	23°28'34.4"N 120°51'55.8"E	2,461	2013/10/11	CKK 8020、CKK 9035、CKK 9047
Hehuanjian	1	24°06'51.4"N 121°13'8.6"E	2,776	2013/09/27	CKS 2002、CKS 2056、CKK 2086
	2	24°08'23.1"N 121°17'13.7"E	3,140	2013/09/27	CKS 5024、CKS 5039、CKK 5099

[1] The collection area of *Cirsium kawakamii* used for isolating endophytic fungi for this study.

Table S2. GenBank strain numbers for phylogenetic tree analysis of the internal transcribed spacer (ITS) and translation elongation factor subunit alpha (EF-1α) sequences in this study

Complex group[a]	Species	Strain number	Accession[b]	
			ITS	EF1-α
--	*Fusarium* sp.	CKK 3008	OR540686	KT273977.1
--	*Fusarium* sp.	CKK 3019	OR540687	KT273978.1
--	*Fusarium* sp.	CKK 4070	OR540688	KT273979.1
--	*Fusarium* sp.	CKK 6059	OR540689	KT273976.1
--	*Fusarium* sp.	CKK 8020	OR540690	KT273983.1
--	*Fusarium* sp.	CKK 9035	OR540691	KT273984.1
--	*Fusarium* sp.	CKK 9047	OR540692	KT273985.1
--	*Fusarium* sp.	CKS 2002	OR540693	KT273986.1
--	*Fusarium* sp.	CKS 2056	OR540694	KT273987.1
--	*Fusarium* sp.	CKS 2086	OR540695	KT273988.1
--	*Fusarium* sp.	CKS 5024	OR540696	KT273989.1
--	*Fusarium* sp.	CKS 5039	OR540697	KT273990.1
--	*Fusarium* sp.	CKS 5099	OR540698	KT273991.1
FTSC 1	*F. gamsii*	NRRL 34036	GQ505451.1	OL772879.1
FTSC 2	*F. acuminatum*	NRRL 13332	OL832225.1	OL772797.1
FTSC 2	*F. acuminatum*	NRRL 13403	OL832175.1	OL772747.1
FTSC 2	*F. acuminatum*	NRRL 13908	OL832226.1	OL772798.1
FTSC 2	*F. acuminatum*	NRRL 25380	OL832242.1	OL772814.1
FTSC 2	*F. acuminatum*	NRRL 28653	OL832231.1	OL772803.1
FTSC 2	*F. acuminatum*	NRRL 28654	OL832249.1	OL772821.1
FTSC 2	*F. acuminatum*	NRRL 36147	OL832308.1	OL772880.1
FTSC 2	*F. acuminatum*	NRRL 53951	OL832232.1	OL772804.1
FTSC 3	*F. tricinctum*	NRRL 26892	OL832254.1	OL772826.1
FTSC 3	*F. tricinctum*	NRRL 36254	OL832262.1	OL772834.1
FTSC 3	*F. tricinctum*	NRRL 36381	OL832258.1	OL772830.1
FTSC 3	*F. tricinctum*	NRRL 36461	OL832255.1	OL772827.1
FTSC 3	*F. tricinctum*	NRRL 53913	OL832259.1	OL772831.1
FTSC 4	*F. avenaceum*	NRRL 26889	OL832176.1	OL772748.1
FTSC 4	*F. avenaceum*	NRRL 36069	OL832186.1	OL772758.1
FTSC 4	*F. avenaceum*	NRRL 36374	OL832183.1	OL772755.1

Complex group[a]	Species	Strain number	Accession[b]	
			ITS	EF1-α
FTSC 4	*F. avenaceum*	NRRL 53589	OL832187.1	OL772759.1
FTSC 4	*F. avenaceum*	NRRL 53729	OL832188.1	OL772760.1
FTSC 4	*F. avenaceum*	NRRL 53733	OL832184.1	OL772756.1
FTSC 4	*F. avenaceum*	NRRL 26890	OL832177.1	OL772749.1
FTSC 4	*F. avenaceum*	NRRL 54939	OL832320.1	OL772892.1
FTSC 5	*Fusarium* sp.	NRRL 52726	OL832312.1	OL772884.1
FTSC 5	*Fusarium* sp.	NRRL 52727	OL832313.1	OL772885.1
FTSC 5	*Fusarium* sp.	NRRL 52730	OL832314.1	OL772886.1
FTSC 6	*F. iranicum*	NRRL 52714	OL832311.1	OL772883.1
FTSC 6	*F. iranicum*	NRRL 52933	OL832317.1	OL772889.1
FTSC 7	*F. flocciferum*	NRRL 25473	OL832306.1	OL772878.1
FTSC 7	*F. flocciferum*	NRRL 40008	OL832325.1	OL772897.1
FTSC 7	*F. flocciferum*	NRRL 45999	OL832310.1	OL772882.1
FTSC 8	*Fusarium* sp.	NRRL 52722	OL832318.1	JF740804.1
FTSC 9	*F. torulosum*	NRRL 22746	OL832295.1	OL772867.1
FTSC 9	*F. torulosum*	NRRL 36514	OL832296.1	OL772868.1
FTSC 9	*F. torulosum*	NRRL 52772	OL832315.1	MH582397.1
FTSC 9	*F. torulosum*	NRRL 36226	OL832178.1	OL772750.1
FTSC 10	*Fusarium* sp.	NRRL 13319	OL832222.1	OL772794.1
FTSC 10	*Fusarium* sp.	NRRL 36132	OL832217.1	OL772789.1
FTSC 12	*Fusarium* sp.	NRRL 36398	OL832270.1	OL772842.1
FTSC 12	*Fusarium* sp.	NRRL 39526	OL832264.1	OL772836.1
FTSC 12	*Fusarium* sp.	NRRL 40096	OL832267.1	OL772839.1
FTSC 13	*F. reticulatum*	NRRL 36339	OL832280.1	OL772852.1
FTSC 13	*F. reticulatum*	NRRL 25792	OL832274.1	OL772846.1
FTSC 14	*F. negundinis*	NRRL 13561	OL832250.1	OL772822.1
FTSC 14	*F. negundinis*	NRRL 20682	OL832181.1	OL772753.1
FTSC 14	*F. negundinis*	NRRL 28287	OL832182.1	OL772754.1
FTSC 16	*F. sinensis*	KOD 941	OL832223.1	OL772795.1
FTSC 17	*Fusarium* sp.	NRRL 53952	OL832281.1	OL772853.1
FTSC 18	*Fusarium* sp.	NRRL 36565	OL832304.1	OL772876.1
FTSC 19	*Fusarium* sp.	NRRL A28022	OL832206.1	OL772778.1
FTSC 21	*Fusarium* sp.	NRRL 25472	OL832207.1	OL772779.1

Complex group[a]	Species	Strain number	Accession[b]	
			ITS	EF1-α
FTSC 21	*Fusarium* sp.	NRRL 28652	OL832209.1	OL772781.1
FTSC 21	*Fusarium* sp.	NRRL 39815	OL832214.1	OL772786.1
FFSC	*F. proliferatum*	NRRL 31071	AF291061.1	AF291058.1
FFSC	*F. fujikuroi*	NRRL 43470	DQ790538.1	DQ790494.1
FFSC	*F. verticillioides*	NRRL 43697	EF453174.1	EF453022.1
FSSC	*F. solani*	NRRL 46643	GU250667.1	GU250544.1
FSSC	*F. falciforme*	NRRL 43441	DQ790522.1	DQ790478.1
FOSC	*F. oxysporum*	NRRL 43542	DQ790553.1	DQ790509.1

[a] FTSC: *Fusarium tricinctum* species complex; FFSC: *Fusarium fujikuroi* species complex; FSSC: *Fusarium solani* species complex; FOSC: *Fusarium oxysporum* species complex.

[b] *Fusarium* internal transcribed spacer (ITS) and translation elongation factor 1 alpha (EF-1α) sequences accession in GenBank.

Table S3. Morphological characteristics of the endophytic *Fusarium tricinctum* species complex (FTSC)-like isolates collected from *Cirsium kawakamii* in this study

Spore	Shape	Media	Size(μm)		Characters on PSA
			Length (mean)	Width (mean)	
Chlamydospore	Globose to subglobose	Soil broth[1]	04.3 – 14.8 (7.8)	0.4 – 3.9 (6.1)	Aerial mycelium abundant and cottony above the agar surface. Produces red pigments. Rare orange sporodochial conidial masses observed.
Macroconidia	Sickle, slight curved, 1-5 septates (most 1 or 3 septate) hooked apical cell and foot-shaped basal cell	SNA[2]	18.7 – 72.5 (35.8)	2.1 – 4.8 (3.7)	
Microconidia	Fusiform, nonseptate	SNA	06.5 – 16.7 (12.0)	1.8 – 4.1 (2.9)	

[1] Soil broth promotes Fusarium chlamydospore formation [70].

[2] Synthetic nutrient agar (SNA) is the medium for the classification of *Fusarium* species [71].
