# Supplementary figures and images for: Endophytic Fungal Diversity in Cirsium kawakamii from Taiwan
Source: J Fungi (Basel). 2023 Nov 3;9(11):1076. doi: 10.3390/jof9111076 (PMC10671896; doi:10.3390/jof9111076)

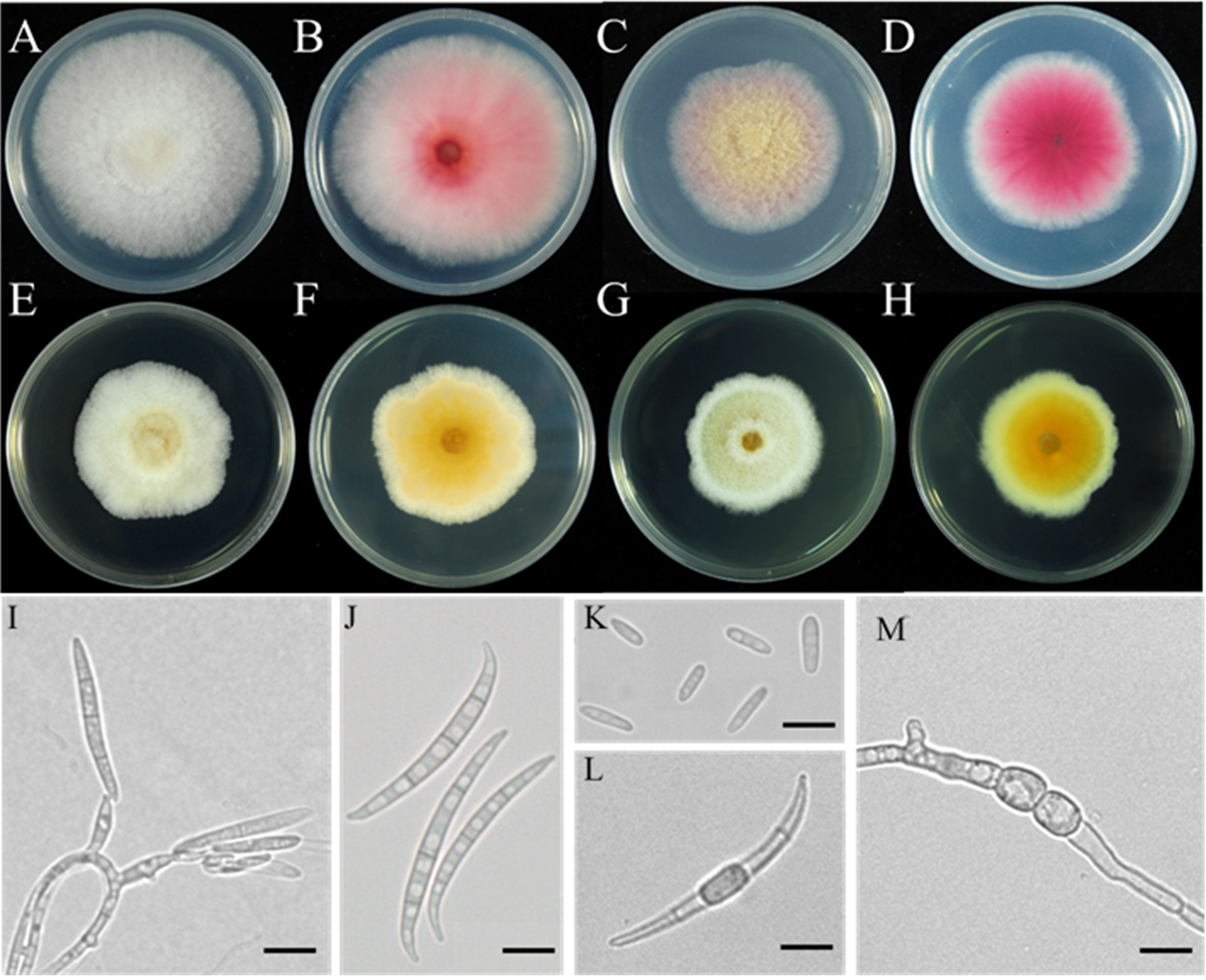

Supplement: Supplementary file 1 [file jof-09-01076-s001.zip › FIG S1.tif]

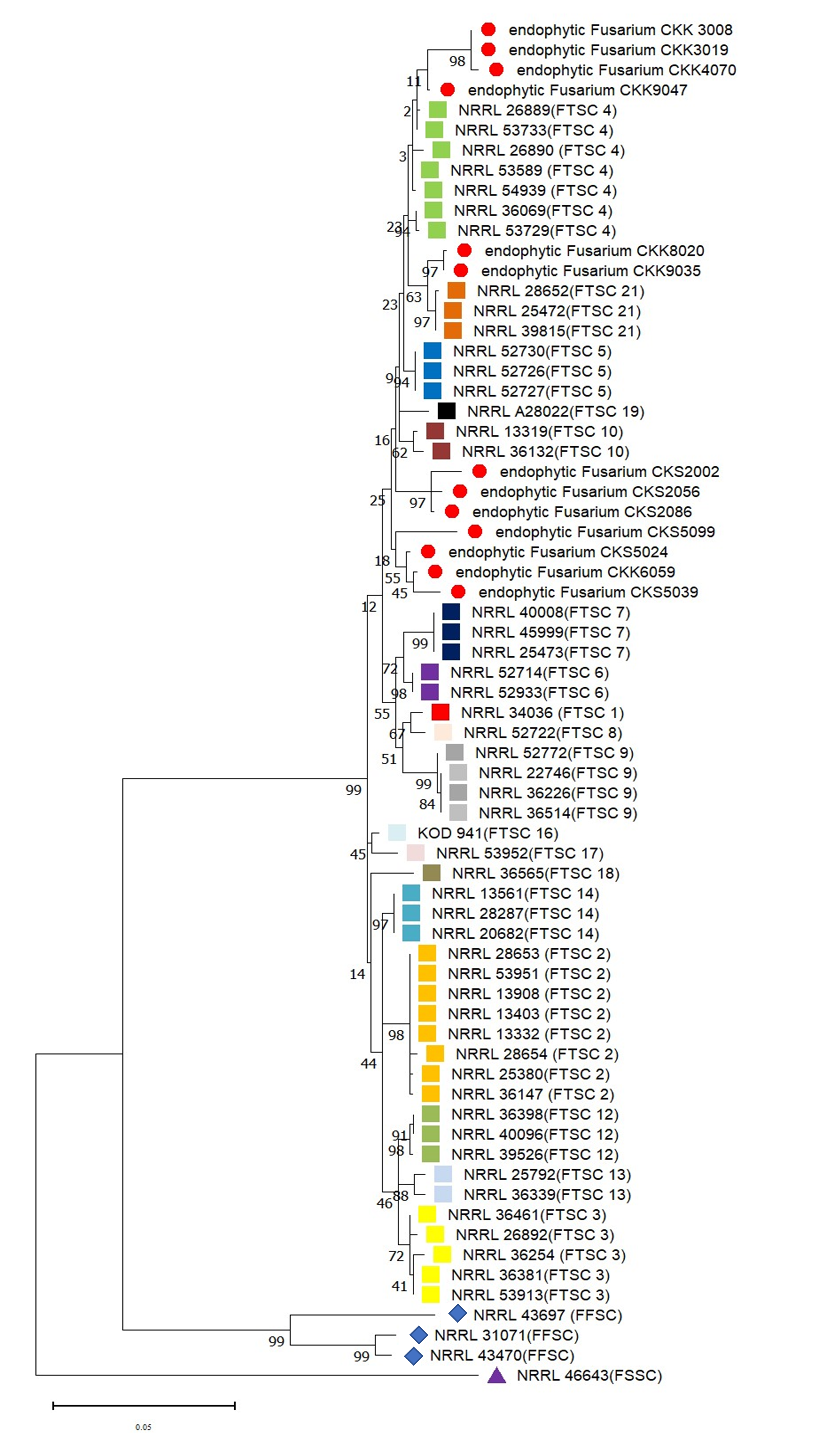

Supplement: Supplementary file 1 [file jof-09-01076-s001.zip › FIG S2.tif]

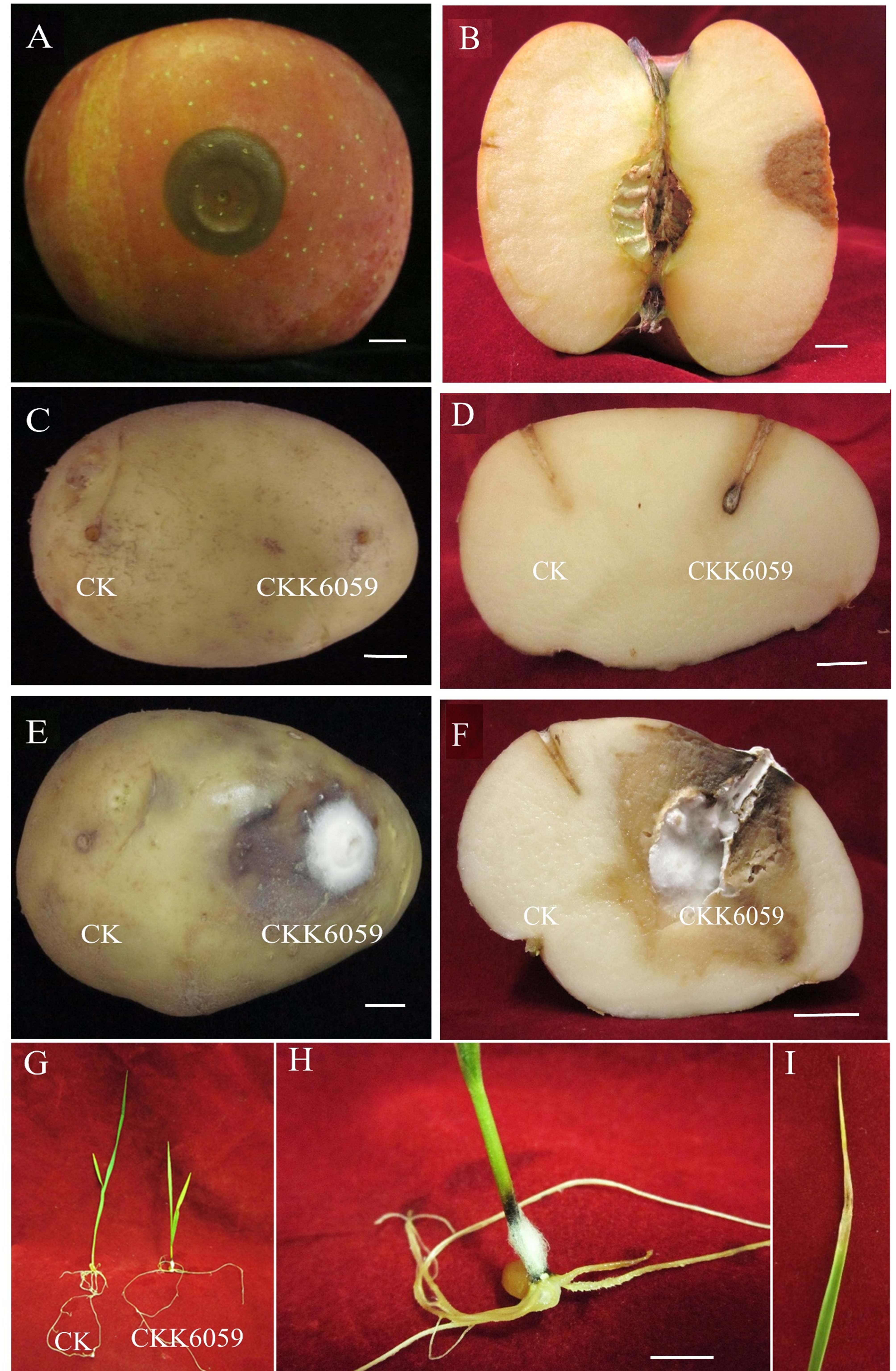

Supplement: Supplementary file 1 [file jof-09-01076-s001.zip › FIG S3.tif]
